# Space‐for‐time substitutions exaggerate urban bird–habitat ecological relationships

**DOI:** 10.1111/1365-2656.14194

**Published:** 2024-11-06

**Authors:** Harold N. Eyster, Kai M. A. Chan, Morgan E. Fletcher, Brian Beckage

**Affiliations:** ^1^ Gund Institute for Environment University of Vermont Burlington Vermont USA; ^2^ Department of Plant Biology University of Vermont Burlington Vermont USA; ^3^ Institute for Resources, Environment & Sustainability University of British Columbia Vancouver British Columbia Canada; ^4^ Biodiversity Research Centre University of British Columbia Vancouver British Columbia Canada; ^5^ Rubenstein School of Environment and Natural Resources University of Vermont Burlington Vermont USA; ^6^ Present address: The Nature Conservancy in Colorado Boulder Colorado USA

**Keywords:** Bayesian inference, longitudinal data, long‐term data, space‐for‐time substitution, urban bird conservation, urban ecology, Vancouver

## Abstract

1. North American bird abundance has declined by 29% over the last 50 years. These continental population dynamics interact with local landscape changes to affect local bird diversity. Mitigating local declines in cities is particularly significant because (a) such declines greatly impact human–bird relationships since most people live in cities and (b) cities provide levers to create bird‐friendly habitat, such as managing yards and gardens, street trees, and urban parks.
2. Yet, the potential for cities to modify habitats to mitigate broader bird declines remains unclear. Studies have been stymied by the difficulty of assembling mutidecadal habitat–bird population datasets. Instead, studies have substituted space for time (e.g. used habitat associations across space at one time point to project future species abundance due to changing land use), but this method may fail amidst nonstationary environments of the Anthropocene.
3. Here, we test the validity of space‐for‐time substitutions for explaining changes in bird abundance in a North American city over the past two decades by examining the degree to which these changes are explainable by changes in local landcover at multiple spatial scales. Specifically, we use longitudinal urban bird surveys of Metro Vancouver, BC, Canada from 1997 and 2020; deep learning models of remote sensing data to classify contemporaneous landcover; out‐of‐sample prediction and boosted regression trees to identify multiple spatial scales of landcover that best explained bird abundance (i.e. optimal scale of effect for each species by each habitat); and Bayesian multispecies abundance models in Stan to determine relationships between changes in landcover and bird abundance.
4. We found that total bird abundance declined by 26% over the last two decades. Landcover measured at both 50 m and optimal scales explained spatial variation in bird abundance, but only landcover at the optimal scale explained temporal changes, and only partially.
5. These results suggest that space‐for‐time substitutions overemphasize habitat–bird ecological relationships, urban habitats only partially determine bird abundance, and measuring habitat at the appropriate scale is important for capturing the most relevant changes in landscapes.

## Graphical abstract

Ecologists often rely on space‐for‐time substitutions to predict future ecosystem changes. Our results suggest that such space‐for‐time substitutions are unlikely to be predictive in the Anthropocene.

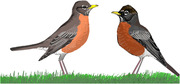

## 1 INTRODUCTION

North American birds have declined by 29% since 1970 (Rosenberg et al., 2019). Very abundant species have experienced particularly large declines (Berigan et al., 2021; Rosenberg et al., 2019). These declines occur amidst climate change, declines in other taxa and urbanization (IPBES, 2019; United Nations Environment Programme, 2021).

Understanding and minimizing these declines is particularly important, and promising, in cities. First, because most people live in cities (United Nations Department of Economic and Social Affairs, 2018), bird population changes in cities may affect human–nature relationships particularly severely. Declines in urban bird populations can diminish human well‐being, erode the positive experiences derived from interactions with nature, and affect the equitability of access to nature (Cox et al., 2017; Langhans et al., 2023; Shanahan et al., 2015). Second, cities offer many opportunities for people to alter local habitat to create landscapes that might better support birds, through actions such as rewilding yards and gardens (Goddard et al., 2010), planting and maintaining particular types of street trees (Wood & Esaian, 2020), creating bird‐friendly habitats in urban parks, and supplementing wild bird diets (Fuller et al., 2007).

Yet, the capacity for cities to mitigate or even reverse bird declines remains inadequately understood due in part to the customary substitution of space for time (Damgaard, 2019), the rarity of long‐term studies and the scale‐dependence of many bird–habitat relationships. Studies on how urban habitats affect birds typically use space‐for‐time substitution (e.g. Coffey et al., 2023; Smallwood & Wood, 2023). Such studies use effects of habitat gradients across space on occurrence as proxies for effects of changes in habitats across time. Space‐for‐time substitution is convenient and can provide insights into the types of habitats preferred by birds (Pickett, 1989). However, the capacity for spatial relationships to be used to predict temporal changes has long been questioned (Damgaard, 2019; Pickett, 1989). Instead, the spatial relationships identified at particular time periods may be due to temporary correlations, rather than mechanistic processes that persist through time (Damgaard, 2019). Given how rapidly greenhouse gas emissions, land‐use change and other factors are restructuring ecological landscapes in the Anthropocene (IPBES, 2019), assumptions of stationarity may no longer hold (Milly et al., 2008), suggesting that predictions from space‐for‐time substitution studies may be particularly inaccurate today (Damgaard, 2019).

Longitudinal studies can help isolate the effects of urban land cover change on bird abundance (Franklin, 1989). Scientists and policymakers have recognized the value of long‐term ecological studies, which may better isolate the effect of land cover than space‐for‐time substitution studies (Hughes et al., 2017). Long‐term ecological studies are essential for testing the inferences from space‐for‐time studies and engaging with the complex causative pathways in ecological systems (Lindenmayer et al., 2012). However, long‐term ecological studies of birds in cities are rare, and even when longitudinal studies are conducted, they often only last for 5–10 years (Fidino & Magle, 2017).

The importance of considering spatial scale has long been recognized (Levin, 1992; Levins, 1968). Studies have shown that habitats affect birds at multiple scales, and that single‐scale models cannot capture the full bird–habitat relationship (Boscolo & Metzger, 2009; Cunningham et al., 2013; Hagen et al., 2016). Recent research has also developed new methods for identifying the optimal scale of effect (Frishkoff et al., 2019; Stevens & Conway, 2019).

Studying urban bird populations—rather than extrapolating from rural populations—is essential. Studies have shown significant rural–urban differences in demography and population dynamics. For example, urban clutch sizes are smaller, and lay dates are earlier (Chamberlain et al., 2009). Nestlings also generally weigh less, suggesting that arthropod food sources (which most landbirds rely on for feeding their young) may be particularly limiting for reproduction in cities (Chamberlain et al., 2009). However, fledglings/pair/year and annual survival (i.e. per cent of birds of various ages/sexes that survive through a year) are often higher in cities (Chamberlain et al., 2009). Urban birds also exhibit substantial phenotypic plasticity in cities, including in their nesting behaviour traits (Bressler et al., 2020). Urban birds are also exposed to different threats, including urban pollutants (Eeva et al., 2000), collisions with glass buildings (De Groot et al., 2021), urban predators (Rodewald, 2011), and diseases from high‐density feeders (Pennycott et al., 2002).

Here, we ask how has bird abundance changed in a North American city over the past two decades, how well do bird–habitat relationships explain these temporal changes (i.e. how valid are space‐for‐time substitutions), and does the scale of landcover measurement matter? To answer these questions, we leverage a longitudinal study of a bird community and land cover measured at a 23‐year interval (in 1997 and 2020) in Metro Vancouver, British Columbia, Canada. We hypothesize that bird abundance has declined, that contemporaneous land cover will explain bird abundance at each timepoint and that temporal changes in land cover will partially predict temporal changes in bird abundance. We hypothesize that land cover may best predict less‐common, less urban‐adapted bird species, and that these predictions will be improved by accounting for multiscale land cover change.

## 2 MATERIALS AND METHODS

### 2.1 Overview

We replicated breeding bird point counts in 2020 that had been originally surveyed in 1997 in Metro Vancouver, British Columbia, Canada. We estimated land cover at each time point using historical RGB aerial imagery, modern satellite imagery, modern land cover annotations, and a deep learning image classification algorithm. We built Bayesian generative multispecies abundance models of the effects of land cover on bird abundance. In the first model, we used land cover of the 50‐m radius point count. For the other models, we used land cover at optimal scales for each species by habitat type, identified using predictive capacity of univariate models or boosted regression trees. We used these multispecies abundance models to simulate bird communities (i.e. posterior communities) and to test the capacity of land cover change to explain change in bird abundance.

### 2.2 Bird community data

**FIGURE 1:**
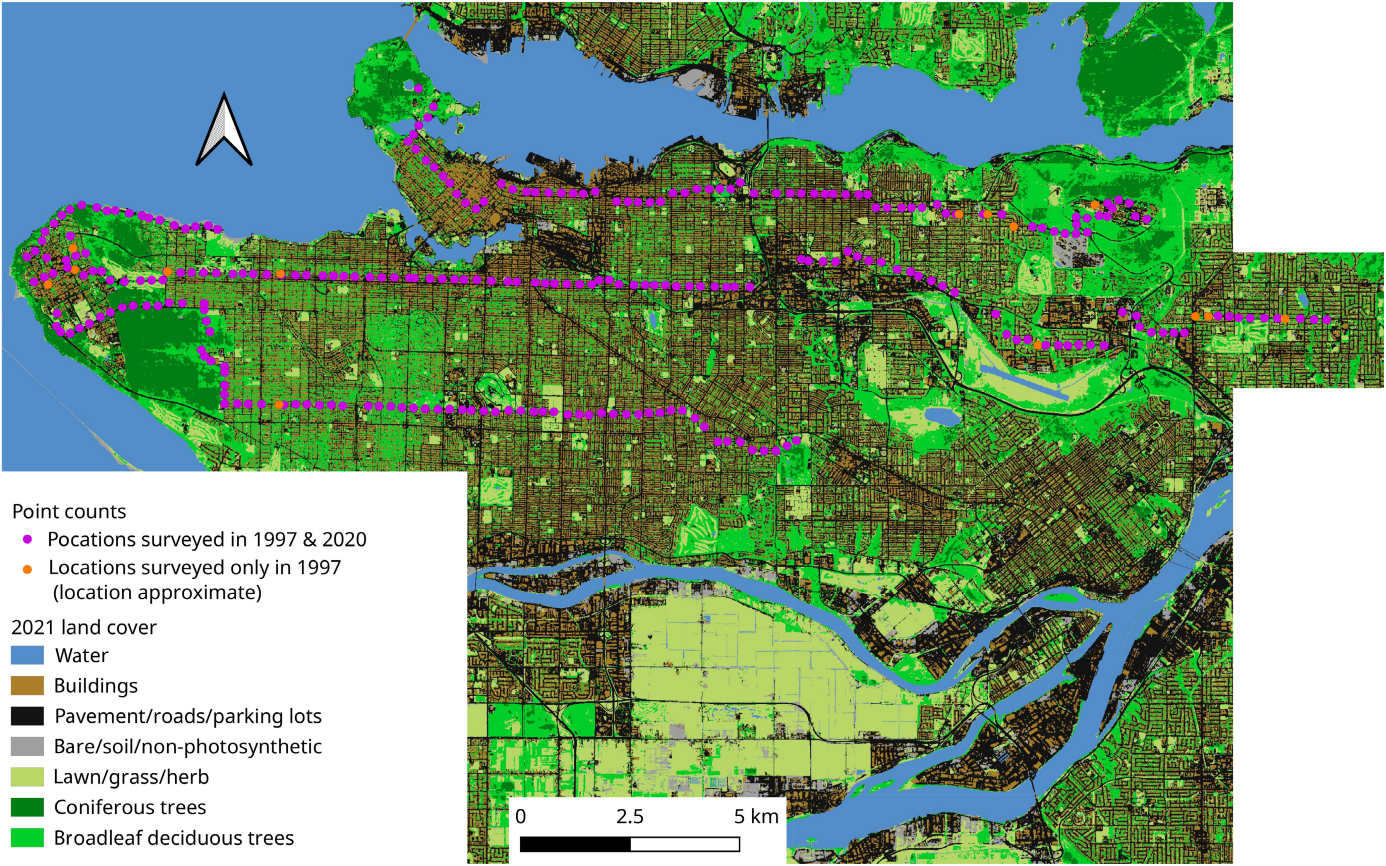
Map of Greater Vancouver, British Columbia, Canada showing 2021 land cover (different colours) and locations of 1997 and 2020 bird surveys (circles; purple circles are replicated surveys, yellow circles are those only surveyed in 1997). For map of historical landcover, see Figure S12.

Original bird surveys were conducted in Metro Vancouver, British Columbia, Canada (Figure 1), by Dr. Stephanie Melles for her Master's Thesis (Melles, 2000) and the data were used in two publications (Melles, 2005; Melles et al., 2003). She surveyed 285 points across the urban region; sites were selected to represent the urban–rural gradient (Melles, 2000). Point counts lasted 5 min, covered 50 m radii, and were only carried out on clear days during the first 4 h following sunrise (Melles, 2000). We used data from point counts conducted in summer 1997 (24 June to 13 July) (designated Period 1). Dr. Melles generously shared these data with us, and they were then organized and archived by the Living Data Project (Bledsoe et al., 2022; Brown et al., 2021). However, only raw data for species seen more than once were available.

We resurveyed these historical point counts in summer 2020 (17 June to 17 July) (designated Period 2). Of the 285 point count locations originally surveyed, we were able to relocate 272 (Figure 1). The first author conducted most surveys; Scott Wilson conducted 19 surveys and Krista De Groot conducted 14 surveys. The resurvey protocol was identical to the historical protocol, except that 5‐min point counts were repeated back‐to‐back and birds were recorded separately during both sampling replicates in order to obtain data necessary to estimate detection probabilities (Dorazio et al., 2006). Only species observed more than once were used for analysis, to match the data available for Period 1. Moreover, we only used Period 1 data from the 272 points count locations that we resurveyed. Data collection was carried out in compliance with the University of British Columbia Animal Care Committee.

To validate that using just two time points is likely to provide meaningful insights, we conducted an analysis of a nearby North American Breeding Bird Survey route (11–212 POINT GREY) that spanned many years (Ziolkowski et al., 2023). We graphically compared trends between just the endpoints (1994, 2018; similar to our timepoints) and the full range of 10 observations between 1994 and 2018. We found that the endpoint trends provided adequate estimates of the full range of data (Figure S1), though trends for some species may not represent many‐year trends.

### 2.3 Land cover covariates

To estimate land cover covariates, we used a deep learning algorithm to classify seven land cover types associated with each period from aerial images, including water, buildings, pavement/roads/parking lots, soil/barren, grass/herb, conifers, and broadleaf trees (for full details, see Eyster & Beckage, 2024). For Period 1 land cover, we obtained 1 m spatial resolution (RGB, 16‐bit unsigned integer) aerial photographs captured over Vancouver, BC, by airplane in 1995. For Period 2, we obtained 1 m spatial resolution RGB satellite imagery taken over Vancouver in 2021 by Maxar Technologies via Google Satellite Imagery (Mountain View, CA; accessed 01 November 2022).

To classify land cover in these images, we used TensorFlow 2 (version 2.11.0), Keras (version 2.11.0), within a Python (version 3.10.9) environment. Briefly, we used a sympatric 2019 land cover classification (Metro Vancouver, 2019) and the 2021 aerial imagery and the deep learning algorithm DeepLabv3+ (Chen et al., 2018) to train an initial model. Then after manually annotating a small selection of 1995 aerial image (about 15 km^2^ using ThRasE plugin; version 22.3.3a) https://github.com/SMByC/ThRasE in QGIS (version 3.28.0), we fine‐tuned the model by training it on the small 1995 annotated region. We used the initial model to predict land cover in 2021 and the fine‐tuned model to predict land cover in 1995. We manually corrected the water classifications since water is easy to manually detect. We used the resultant raster images to represent landcover at each timepoint (Figure 1 and Figure S12). We used PyQGIS to calculate the proportion of each land cover type at 30 different scales around each point count centroid, ranging from 50 m radius to 1500 m radius, with a step size of 50 m (Frishkoff et al., 2019).

### 2.4 Scale selection

We tested three different methods for determining the optimal scale of effect of each land cover type on each species. These included boosted regression trees and two out‐of‐sample prediction analyses using generalized linear models (one with Pearson correlations and one with Spearman's rank correlations).

#### 2.4.1 Boosted regression trees

Boosted regression trees (BRTs) are flexible and powerful tools for identifying the best predictors of a response (Elith et al., 2008). We built boosted regression trees for each species that included all habitats (*n* = 7) and all scales (*n* = 30) using the gbm (v. 2.1.9) package in R (Ridgeway & Developers, 2024). To determine the best tree fitting parameters, we used the caret package (v. 6.0.94) to test a range of tree parameters on the American Crow data, including number of trees (1000, 5000, 7500, 10,000 or 20,000), shrinkage (0.0001, 0.0005 or 0.001), and number of minimum observations in terminal nodes (5, 10, 15, 20 or 25). Using root mean squared error (RMSE), we selected the best fitting parameters, including number of trees = 20,000, shrinkage = 0.0001, and number of minimum observations in node = 25. We also used bag fraction = 0.5, interaction depth = 1, and cross‐validation folds = 10. We used these tuned parameters to then fit trees for each species and then selected the scales for each land cover that had the highest computed relative influence for explaining the abundance for a given species. We were unable to build trees for species with very few observations; for these species, we used the optimal scales from a closely related species. This analysis resulted in 61 (number of species) × 7 (number of land cover types) = 427 optimal scales, which we then used in Bayesian multispecies abundance models (see Section 2.5. below).

#### 2.4.2 Out‐of‐sample prediction

Stevens and Conway (2019) found that habitat scales selected based on out‐of‐sample predictions (rather than information criteria such as AIC) yielded more predictive multiscale models (i.e. optimal spatial scales for a given habitat are best identified through out‐of‐sample predictive scale selection). We expanded upon Stevens and Conway (2019) to multispecies communities by building univariate models for the abundance y of each species i at sampling location j at within‐period temporal replicate k during period t for each land cover type m and each scale c (ranging from 50 to 1500 m radii) (for definitions of all parameters, see Table 1). Note that because the Period 2 contained within‐period temporal replicates, we used the maximum raw count across these temporal replicates in Period 2 (because the temporal maximum better represents the number of birds associated with the surrounding land cover), but not in Period 1:

(1) $$\max_{k\epsilon \left\{1,2\right\}}y_{\text{ijkmc},t=2}∼\text{Poisson}\;\left(\exp\left(\zeta_{\mathrm{imc},t=2}+\eta_{\mathrm{imc},t=2}E_{\mathrm{jmc},t=2}\right)\right)$$

(2) $$y_{\text{ijmc},t=1}∼\text{Poisson}\left(\exp\left(\zeta_{\mathrm{imc},t=1}+\eta_{\mathrm{imc},t=1}E_{\mathrm{jmc},t=1}\right)\right)$$

where E is the land cover, ζ is the intercept parameter, and η is the slope parameter. We fit these univariate models using the frequentist glm function, due to the volume of models required and the speed of frequentist methods. To test which scales of each land cover type were most predictive for each species, we applied leave‐one‐out cross‐validation for each of the above models (Liu, 2015) (i.e. for each of the 30 scales c, for each of the seven land cover type m and for each of the 61 species i, meaning that we tested 30 × 7 × 61 = 12,810 models). We then calculated Pearson correlation coefficients between the observed data (from both periods) and each of the leave‐one‐out predictions (for both periods) for each land cover type at each scale for each species (we also tested using Spearman's correlation; Montesinos López et al., 2022). We selected the optimal scale for a given species and land cover type by choosing the scale that maximized this correlation. This procedure produced 61 (number of species) × 7 (number of land cover types) = 427 optimal scales, which we used in Bayesian multispecies abundance models. In addition, we also created a model using just the 50 m scale for all species and all land cover covariates to identify the effects of local land cover alone.

**TABLE 1:** Parameters and definitions used in equations in this paper, listed in order of use.

Parameter	Definition
*y*	Measured abundance
Subscript *i*	Identifies species
Subscript *j*	Identifies sampling location
Subscript *k*	Identifies within‐period temporal replicate (only relevant for Period 2)
Subscript *t*	Identifies sampling period (either Period 1 = 1997 or Period 2 = 2020)
Subscript *m*	Identifies land cover type
subscript *c*	Identifies spatial scale at which the land cover is measured (ranges from 50 to 1500 m radii, with 50 m intervals)
E	The proportion of area defined by a given spatial scale covered by a given land cover type during a given sampling period
ζ	Intercept parameter in the generalized linear models used for scale selection
η	Slope parameter for effect of a given land cover at a given scale in the generalized linear models used for scale selection
N	True abundance (i.e. accounting for nondetection)
P	Detection probability
a	First parameter describing the Beta hyperprior used to partially pool species‐specific detection probabilities
b	Second parameter describing the Beta hyperprior used to partially pool species‐specific detection probabilities
ψ	Centre parameter of Beta hyperprior used to partially pool species‐specific detection probabilities
θ	Shape parameter of Beta hyperprior used to partially pool species‐specific detection probabilities
λ	The expectation of the Poisson distribution used to model true abundance
M	The number of land cover types (7)
β	The effect of a given land cover on the expected log abundance of a given bird species in the multispecies abundance model
μ	The mean of the Normal hyperprior from which each bird's β for a given land cover type is drawn
τ	The standard deviation of the Normal hyperprior from which each bird's β for a given land cover type is drawn
ϕ	The log temporal change in abundance of a given species not due to changes in land cover
ρ	The mean of the Normal hyperprior from which each bird's ϕ is drawn
σ	The standard deviation of the Normal hyperprior from which each bird's ϕ is drawn
α	The intercept parameter for a given species in the multiscale multispecies abundance model
$\mu_{\alpha }$	The mean of the Normal hyperprior from which each species' α is drawn
$\tau_{\alpha }$	The mean of the Normal hyperprior from which each species' α is drawn
J	The total number of sampling locations

### 2.5 Multispecies abundance models

Bayesian multispecies abundance models account for imperfect detection and enable data from abundant species to help inform parameters and abundances of rare species (Yamaura et al., 2012). These models produce estimates of ‘true’ abundance, where true designates that this is the abundance after accounting for incomplete detection (Kéry & Royle, 2016). Here, we built four multispecies abundance models. The first uses just the land cover within 50 m of each sampling location, while the next three models use land cover measured at the optimal scales as identified by boosted regression trees, out‐of‐sample predictions and Pearson correlations, and out‐of‐sample predictions and Spearman's correlations, respectively (see above). We then used the posteriors produced by these models to estimate the true abundance in 1997 and 2020. Finally, we tested the validity of the space‐for‐time substitution by using the model posteriors to assess the degree to which land cover relationships can explain temporal changes in bird abundances.

We built multispecies abundance models using a Binomial distribution.

(3) $$y_{\mathrm{ij},t=1}∼\text{Binomial}\left(N_{\mathrm{ij},t=1},P_{i,t=1}\right),$$

(4) $$y_{\mathrm{ijk},t=2}∼\text{Binomial}\left(N_{\mathrm{ij},t=2},P_{i,t=2}\right),$$

(5) $$P_{i,t=1}=P_{i,t=2},$$

(6) $$P_{i,t=2}∼\text{Beta}\left(a,b\right),$$

(7) $$\psi =\frac{a}{a+b},$$

(8) θ=a+b,

where N is the true abundance and P is the detection probability (t is the sampling period and k is within‐period temporal replication; see above and Table 1 for full definitions of subscripts). Because we only had within‐period temporal replicates necessary for estimating detection probability for Period 2, we assumed that detection probabilities were constant across periods. We used a Beta hyperprior for the distribution of detection probabilities of each species (Dorazio & Royle, 2003; Gomez et al., 2018), defined by a centre parameter ψ and a scale parameter θ (Gelman et al., 2013). We used a Beta prior for the detection probability centre parameter and a gamma prior for the scale parameter (Kruschke & Vanpaemel, 2015).

(9) $$\psi ∼\text{Beta}\left(2,3\right),$$

(10) $$\theta ∼\text{Gamma}\left(9,5\right).$$

We modelled N with a Poisson error distribution:

(11) $$N_{\mathrm{ijt}}∼\text{Poisson}\left(\lambda_{\mathrm{ijt}}\right),$$

where λ is the Poisson parameter. For the model with land cover covariates measured at the 50 m radius, we used a log‐linear combination of sampling site‐specific covariates (Royle & Dorazio, 2008).

(12) $$\log\left(\lambda_{\mathrm{ij},t=1}\right)=\sum_{m=1}^{M}\beta_{\mathrm{im}}E_{\mathrm{jm},t=1},$$

(13) $$\beta_{\mathrm{im}}∼\text{Normal}\left(\mu_{m},\tau_{m}\right),$$

(14) $$\log\left(\lambda_{\mathrm{ij},t=2}\right)=\phi_{i}+\sum_{m=1}^{M}\beta_{\mathrm{im}}E_{\mathrm{jm},t=2},$$

(15) $$\phi_{i}∼\text{Normal}\left(\rho ,\sigma \right),$$

where $\beta_{\mathrm{im}}$ is the log expected abundance of species i if land cover type m covered the entire 50 m radius count circle. Because the land cover proportions add to one, including an intercept term creates a predictor matrix that is rank‐deficient. We thus omitted the intercept term, adopting the Scheffe parametrization (Lawson & Willden, 2016; Scheffé, 1958). We partially pooled the effects of a given land cover type on each species' abundance ($\beta_{\mathrm{im}}$) using a Normal distribution hyperprior (Equation 13). $E_{\mathrm{jmt}}$ is the proportion of land covered by land cover type m around sampling point j during period t. For period t=2, we added an additional parameter ϕ which accounts for changes in abundance of species i that is not due to changes in land cover (adapted from Montaño‐Centellas et al., 2020). We treated ϕ as a random effect drawn from a Normal distribution. This pooling helps to inform the parameter values for rarely sampled species. We used Normal priors for μ, τ, ρ and σ.

For the model using covariates at the optimal scales, we modified some of the above equations to include an intercept, α, (because the multiscale model is full rank, since when measured at different scales, land covers do not sum to unity) and species‐dependency to the land cover covariate:

(16) $$\log\left(\lambda_{\mathrm{ij},t=1}\right)=\alpha_{i}+\sum_{m=1}^{M}\beta_{\mathrm{im}}E_{\mathrm{ijm},t=1},$$

(17) $$\alpha_{i}∼\text{Normal}\left(\mu_{\alpha },\tau_{\alpha }\right),$$

(18) $$\log\left(\lambda_{\mathrm{ij},t=2}\right)=\phi_{i}+\left(\alpha_{i}+\sum_{m=1}^{M}\beta_{\mathrm{im}}E_{\mathrm{ijm},t=2}\right),$$

where $E_{\text{ijmt}}$ is the proportion of land covered by land cover type m at sampling point j during period t that is measured at the spatial scale that is optimally predictive for species i. We treated $\alpha_{i}$ as a random effect drawn from a Normal distribution.

We fit al models in Stan version 2.31.0 using cmdstan (Carpenter et al., 2017) with four chains, each with 1500 warmup (discarded) and 500 sampling iterations. We validated model convergence using $\hat{R}$ (Vehtari et al., 2019) and fit using posterior retrodiction checks (Betancourt, 2020). We fit the models using an iterative process, beginning with very simple models, reparametrizing to achieve convergence, then adding additional complexity and repeating until the desired complexity was attained. We conducted initial data wrangling, optimal scale of effect identification and post‐model processing in R (v. 4.2.2).

To estimate the true abundance of each species, we used the models to simulate posterior communities. To calculate the degree to which change in land cover predicts change in bird abundance, we used the fitted parameters to calculate the expected abundance of each species in Period 2 if ϕ=0, that is if bird abundances could only change between Period 1 and Period 2 in response to changes in land cover:

(19) $$\text{Expected}\left(N_{i,t=2,\phi =0}\right)=\sum_{j=1}^{J}\left(\text{Poisson}\left(\exp\left(\alpha_{i}+\sum_{m=1}^{M}\beta_{\mathrm{im}}E_{\mathrm{ijm},t=2}\right)\right)\right).$$

Equation 19 is for the optimal scale model; for the 50 m scale model, we dropped the α term and the species‐dependence of E (i.e. the species‐specific optimal scale). To estimate percent change in both true abundance and abundance when only accounting for land cover change, we replaced zeros in Period 1 (1997) posterior communities with $1\times 10^{-10}$ and then calculated the per cent change between 1997 and 2020.

If space‐for‐time substitution is valid, then we would expect that the bird abundances in Period 2 estimated by Equation 19 would match the bird abundances in Period 2 estimated by the full multispecies abundance model.

To compare Metro Vancouver changes to national changes, we calculated the change in abundance for each species and of all species over the 23‐year period ending in 2016 (the latest date available) using data from the 2019 Canada State of the Birds Report (North American Bird Conservation Initiative Canada, 2019) (updated: 20 June 2019 at 4:50 PM). Most of these data come from the North American Breeding Bird Survey (Sauer et al., 2017).

## 3 RESULTS

**FIGURE 2:**
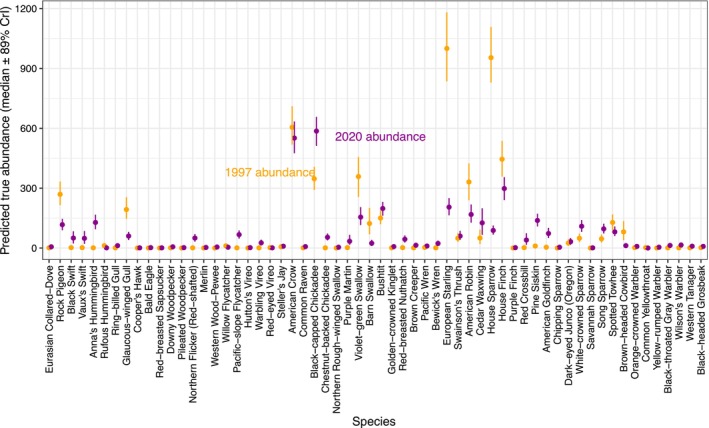
Estimated true abundance of each species across all sampling sites in 1997 (orange) and 2020 (purple) based on the multiscale model using boosted regression trees to estimate optimal spatial scales. Medians and 89% credible intervals are shown. For greater detail for trends in low‐abundance species, see Figure S4.

**FIGURE 3:**
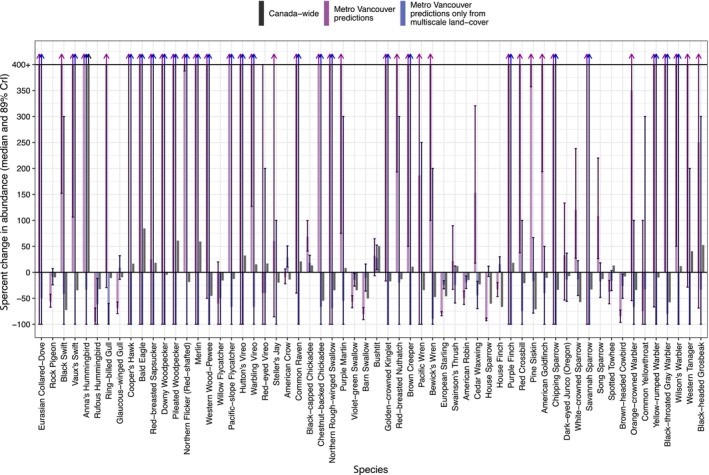
Median per cent change in abundance (±89% credible interval) of each species. The grey bars represent observed abundance across Canada 1993–2016 (data from North American Bird Conservation Initiative Canada, 2019); purple bars represent predicted abundance across greater Vancouver 1997–2020 based on the multiscale model using scales selected by boosted regression trees; blue bars represent predictions based on the same multiscale model if all changes in abundance were completely accounted for by changes in multiscale land cover. If changes in urban abundance were due to nationwide trends, we might expect the purple bars to roughly match the grey ones; if space‐for‐time substitutions were valid, we would expect blue bars to match purple bars. Increases greater than 400% are indicated by arrows pointing above the solid horizontal line at +400%. Multiscale model based on optimal scales selected using boosted regression trees; for model results from other scale selection procedures, see Figures S10 and S11. Note that data for Eurasian Collared‐Dove were not available across Canada. National trends for American Crow are given by national trends for Northwestern Crow. Note that medians and error bars extending beyond 400% are indicated with an arrow.

Model estimates of detections were similar to observed detections (Figures S2 and S3). Estimates of true abundance in 2020 were 26% lower than estimates of true abundance in 1997 (scale‐optimized model, median = 26.0, 89% CrI = 20.1–31.3; Figure 2, Table S4). This Metro Vancouver decline is consistent with the national decline of 31% in the 23‐year period ending in 2016. In addition to changes in total abundance, the dominant species also changed between years. The five most common species in 1997 were the European Starling, House Sparrow, American Crow, House Finch, and Violet‐green Swallow (Figure 2). The five most common species in 2020 were Black‐capped Chickadee, American Crow, House Finch, European Starling, and Bushtit (Figure 2). Some of these species trends were consistent with national trends (e.g. Anna's Hummingbird and American Crow), while other local trends were opposite to national trends (e.g. Cedar Waxwing and spotted Towhee) (Figure 3). These estimates of abundance and abundance change were consistent across all the models (Figure 2, Figure S5).

Both the 50 m and scale‐optimized models revealed strong spatial relationships among each species' abundance and surrounding landcover (Figures S8 and S7). Different species were more abundant in different habitats. For example, Glaucous‐winged Gull and American Crow were more abundant in areas covered by buildings, whereas American Robin and Black‐capped Chickadee were more abundant in areas dominated by deciduous trees. Some species exhibited more generalist habitat preferences, such as American Robin, which was fairly abundant across four land cover types. In contrast, other species only exhibited substantial abundance in one habitat, such as Golden‐crowned Kinglet in conifers (Figure S7).

The scale at which land cover was measured affected the temporal predictive power of land cover through time (i.e. when testing space‐for‐time substitution validity by examining whether temporal changes in bird abundance can be explained by *only* temporal changes in land cover). Two‐decade changes in land cover within 50 m of sampling points poorly explained two‐decade changes in bird abundance (blue bars differ from purple bars in Figure S9). However, two‐decade changes in land cover measured at the optimal scale of effect partially explained changes in some species (blue and purple bars in Figure 3 are more similar), for example for Black‐capped Chickadee and American Robin (Figure 3). Despite each optimal scale identification method producing different optimal scales (Tables S1–S3), the multiscale model using scales selected via boosted regression trees and that using scales selected via Pearson correlations of leave‐one‐out predictions produced similar estimates of two‐decade trends in bird abundances for many species, but not all. For example, the Pearson method better explained Barn Swallow trends (Figure S10), while the regression tree method better explained European Starling trends (Figure 3). However, using Spearman's rank correlations produced worse predictions (Figure S11).

Based on the model results from the boosted regression tree method (Figure 3), we found that 3% of species' predictions were in opposite direction of the real trends (i.e. trends [purple] were opposite of the trends predicted if just using land cover change [blue], based on 89% credible intervals; e.g. Cedar Waxwing), 44% of species showed real trends that were either positive or negative while predictions were not statistically significant, or vice versa (e.g. House Sparrow), 41% of species had both statistically nonsignificant real and predicted trends (e.g. Steller's Jay), 8% of species had significant trend in the same direction (e.g. American Robin), and finally one species (2% of species; Rufous Hummingbird) had significant trends in the same direction with overlapping 89% credible intervals.

## 4 DISCUSSION

Metro Vancouver's bird community has changed substantially over the last two decades, with huge losses in some species (e.g. House Sparrow, European Starling, and Barn Swallow), but gains in others (Anna's Hummingbird, White‐crowned Sparrow; Figure 3). Overall, we found a 26% decrease in bird abundance between 1997 and 2020, just slightly less than the national decline of 31%.

We found low support for the validity of space‐for‐time substitutions. Although we found strong relationships between each bird species' abundance and land cover across time (Figure S7), these bird–habitat relationships only partially explained bird temporal trends, and only for a few species (Figure 3). Moreover, the fact that these trends were explained to a similar degree by the two top multiscale models (Figure 3, Figure S10) despite these models using land cover at different scales (Tables S1 and S2) further suggests that these land cover relationships may be phenomenological rather than mechanistic through time. Statistical patterns across space do not necessarily indicate mechanistic relationships that persist through time. These findings suggest that local habitat changes may not be as important as typically assumed (WWF, 2016) and that ecology's reliance on space‐for‐time substitution instead of longitudinal studies (Damgaard, 2019) may be misplaced. While such space‐for‐time substitution studies provide low‐cost and smaller data requirements, our results suggest that their implications for how habitat modifications can support populations may be overstated. A recent study of forest and open‐habitat birds found that spatial and temporal habitat changes produced similar overall changes in species richness and total abundance (Attinello et al., 2023); perhaps our study did not find these similarities because space‐for‐time substitutions are particularly inappropriate in urban landscapes, or because any similarities disappear when examining species‐level trends. Long‐term studies like ours may be essential for unearthing the true relationships between habitat and birds, and showing the limited power of urban spatial relationships to predict change through time. Our results suggest that space‐for‐time substitutions ought to be interpreted with caution and not be employed to predict far‐future changes in urban bird abundances or distributions. For example, our results cast doubt on reports like the National Audubon Society's Survival by Degrees report, which relied on space‐for‐time substitutions to predict future range changes of particular bird species in response to future climate change (Bateman et al., 2020; Wilsey et al., 2019).

The scant capacity for spatial relationships with habitat to explain temporal changes in bird abundance may be due to a number of factors, such as interspecific interactions, regional species pool effects, changes in arthropod abundance, climate change, microhabitat specificity, and changes in anthropogenic food subsidies. First, for example, we found large declines in European Starling abundance, but increases in Norther Flicker (Red‐shafted) abundance (Figure 3). Studies have shown that starlings limit flicker nesting success (Turner et al., 2004), suggesting that starling trends may have driven flicker trends, as well as flicker habitat preferences through relaxation of competitive exclusion. Similarly, House Sparrows may also be declining due to Cooper's Hawk increases (Bell, 2011). Future research might explicitly model the community‐specific nature of habitat preferences (Eyster et al., 2023) and also account for interspecific interactions and indirect effects. We employed univariate random effects to account for correlations among species' responses, but future studies might employ latent variable models (LVMs) to examine the relative effects of unmeasured latent variables and land cover variables (Warton et al., 2015).

Second, the regional species pool may also swamp the effects of local habitat changes through mass effects (Brawn & Robinson, 1996; Leibold et al., 2004). That is, even if local habitats change to become very inhospitable to a species, that species may remain abundant because of net dispersal from an external, stable species pool. For example, we found that some species' trends matched national trends (Figure 3), suggesting that local conservation efforts must be complemented by regional and international efforts. It is also possible that there are time lags in the effects of habitat on abundance that our study was too short to assess (Chen et al., 2023), and that mass effects may be moderated by species‐specific functional traits related to dispersal ability, and could be tested by future work (Eyster et al., 2022).

Third, changes in arthropod abundance may drive bird abundance changes (Tallamy & Shriver, 2021). Two‐decade declines in insects have been shown to be associated with the rate that birds feed their young and bird abundance in Denmark (Møller, 2019) and associated with bird declines in Europe more broadly (Bowler et al., 2019; Wagner, 2020). Such arthropod declines may cause changes in bird diets and may induce bird declines. For example, shifts in Vaux's Swift diet have been associated with population declines (Pomfret et al., 2014). North American arthropod data are scarce (Wagner, 2020), and British Columbian arthropod data are scarcer, but surveys suggest that household use of pesticides (both herbicides and insecticides) in British Columbia has declined since 1994 (Environment and Climate Change Canada, 2021a); more recent data (2007–2019) on insecticide use in BC suggest that insecticide use has also declined slightly (Statistics Canada, 2021). However, neonicotinoid pesticides were first authorized for use in the United States in 1994 and Canada in 1995 and have been shown to cause mortality and other harms to birds (Eng et al., 2017; Humann‐Guilleminot et al., 2019; Mineau & Stephanie von Blackwood, 2013). Our understanding of arthropod changes, and their effects on birds, is low, particularly in British Columbia. Future research might combine arthropod and land cover trends to better understand and predict changes in bird abundance.

Fourth, climate change may play a role in these avian trends. Climate change is affecting bird phenology (Torti & Dunn, 2005), and phenological mismatches may lead to lower fitness (though there is little evidence of effects of mismatches on population declines; Samplonius et al., 2020). As spring advances, bird breeding phenology is not keeping up; for every 4 day advance in vegetation, bird breeding only advances by 1 day, with negative implications for breeding productivity (Youngflesh et al., 2023). Climate change may also directly kill birds through heat waves—which Metro Vancouver has recently experienced (Raymond et al., 2022)—and water stress (McKechnie & Wolf, 2009), and anthropogenic forest fires may affect bird foraging (Sanderfoot et al., 2022). Climate change may also indirectly affect birds via effects on arthropod prey. For example, a study of beetles in southern British Columbia found that wild‐caught beetles have declined in size and that this decline is associated with increased temperatures due to climate change (Tseng et al., 2018). Declines in prey size may increase the energy costs of foraging. Accounting for climate change is important for forecasting future urban bird populations and how to support them.

Fifth, while our study did differentiate between conifers and broadleaf trees, we were not able to distinguish between tree genera or species. It is possible that our land cover categories were too general to capture all the meaningful changes in habitats. For example, some particular species of trees are much more attractive to birds than others (Wood & Esaian, 2020). Modern satellite imagery with high spectral and spatial resolutions and LiDAR data may help distinguish among these tree types in future studies (Wang et al., 2019).

Sixth, anthropogenic food subsidies and other human actions may have had an outsized effect on bird abundances. Many of the species that showed the greatest losses are relatively recent arrivals to North America that have very strong relationships with people, such as Rock Pigeon, European Starling and House Sparrow (Ravinet et al., 2018). These species may also be more sensitive to changes in human food subsidies and other social factors (Moulton et al., 2010), and many of their trends were poorly explained by land cover changes, as hypothesized (Figure 3). While such species are often treated as detrimental to biodiversity and native bird communities (Bellard et al., 2016; Fisher & Wiebe, 2005; cf. Biermann & Mansfield, 2014; Davis et al., 2011), these species are urban‐adapted and are most common in the most built‐up parts of Metro Vancouver (Figure S7; Ravinet et al., 2018). Thus, these species may provide key ways for people to access nature in the most urbanized parts of cities, and their decline may limit human interactions with wildlife (Soga & Gaston, 2016; Turner et al., 2004) and lead to the so‐called ‘extinction of experience’ (Gaston & Soga, 2020).

Accounting for habitat change at the scale of effect proved essential for predicting some changes in bird abundance through time. Our models that included each land cover measured at the optimal scale for each species better explained temporal changes in bird abundance than the model that only accounted for land cover measured at the local 50 m scale (Figure 3, Figure S9). This importance of accounting for habitat at multiple scales is consistent with previous research (Boscolo & Metzger, 2009; Cunningham et al., 2013; Hagen et al., 2016; Stevens & Conway, 2019).

Our survey method has some limitations; it was not designed to measure waterbirds such as Canada Goose (i.e. surveys were not located near water bodies), so trends in those species are likely not informative (we thus removed Canada Goose from main text figures). Additionally, two timepoints are likely not informative of population dynamics of irruptive species, including Red‐breasted Nuthatch, Pine Siskin and Red Crossbill (Cottee‐Jones et al., 2015). Our secondary analysis of North American Breeding Bird Survey trends suggests that two timepoints are adequate for most species. Our estimates of change are also only for breeding birds observed in the summer; migratory species and overwintering species may show other trends and other relationships with land cover. Future work might extend this analysis to birds throughout the year.

Beyond the theoretical implications for space‐for‐time substitutions in ecology, our results also have management implications for North America's birds. Vancouver has plans to increase its tree cover to 22% by 2050 (the City of Vancouver and Vancouver Park Board, 2018), in part to address heat waves (Eyster & Beckage, 2022, 2023) and Canada has plans to plant 2 billion trees (Environment and Climate Change Canada, 2021b). Our results suggest that careful tree planting and habitat modification of urban habitat may help boost local abundance of some birds (e.g. replacing impervious surfaces with greenscapes may increase American Robin populations; Figure S8), but that birds are also likely responding to interspecific interactions, regional species pools, arthropods abundance, climate change, specific microhabitats and anthropogenic food resources. These other direct and indirect drivers are likely important and must be accounted for in tandem with local habitat restoration. Nevertheless, our results show that different types of habitats may support different birds, and our results may help inform tree planting in Metro Vancouver (Figures S7 and S8).

## AUTHOR CONTRIBUTIONS

Harold N. Eyster and Kai M. A. Chan conceived the ideas and designed the methodology; Harold N. Eyster collected the data; Morgan E. Fletcher transcribed data; Harold N. Eyster analysed the data and created visualizations; Brian Beckage contributed to model development and analysis; Kai M. A. Chan and Brian Beckage contributed to visualization design; Harold N. Eyster led the writing of the manuscript. All authors contributed critically to the drafts and gave final approval for publication. Kai M. A. Chan and Harold N. Eyster lived in the location where the data were collected and their knowledge of, and concern for, Vancouver's biodiversity shaped this study. The study was designed and conducted in consultation with local people.

## CONFLICT OF INTEREST STATEMENT

The authors declare no conflicts of interest.

## STATEMENT OF INCLUSION

Harold N. Eyster and Kai M. A. Chan resided in the city in which the data was collected.

## Supporting information


**Table S1.** Optimal scales for each species and each land cover type found using boosted regression trees.
**Table S2.** Optimal scales for each species and each land cover type found using out‐of sample prediction and Pearson correlations.
**Table S3.** Optimal scales for each species and each land cover type found using out‐of sample prediction and Spearman's correlations.
**Table S4.** Estimates of true abundance (medians and 89% CrI).
**Figure S1.** Plots of birds contained in our datasets that were also detected by the North American Breeding Bird Survey route 11‐212.
**Figure S2.** Total observed (black dots) and modeled (orange circles and 89% credible intervals) detections of each species in 1997.
**Figure S3.** Total observed (black dots) and modeled (purple circles and 89% credible intervals) detections of each species in 2020.
**Figure S4.** Estimated true abundance of less‐abundant species across all sampling sites in 1997 (orange) and 2020 (purple) based on the multiscale model using boosted regression trees to estimate optimal spatial scales.
**Figure S5.** Estimated true abundance of each species across all sampling sites in 1997 (orange) and 2020 (purple) based on the 50‐m model.
**Figure S6.** Estimated true abundance of each species if the entire 50‐m area surrounding each sampling point consisted of the indicated habitat type in 1997 (orange) and 2020 (purple).
**Figure S7.** Modeled abundance of each species in 1997 if the entire 50‐m site was occupied by the indicated land cover type.
**Figure S8.** Estimated effects of each habitat type when measured at the optimum scale (identified using boosted regression trees) for each species in 1997 (orange) and 2020 (purple).
**Figure S9.** Percent change in abundance of each bird species across greater Vancouver 1997–2020 based on the 50 m landcover model (purple) and based on the 50 m landcover model if all changes in abundance were completely accounted for by changes in landcover within the 50‐m radius point count (blue).
**Figure S10.** Percent change in abundance of each bird species across Canada 1993–2016 (gray; data from North American Bird Conservation Initiative Canada (2019)), across greater Vancouver 1997–2020 based on the multiscale model using Pearson correlations (purple) and based on the multiscale model (again using Pearson correlations) if all changes in abundance were completely accounted for by changes in multiscale land cover (blue).
**Figure S11.** Percent change in abundance of each bird species across Canada 1993–2016 (gray; data from North American Bird Conservation Initiative Canada (2019)), across greater Vancouver 1997–2020 based on the multiscale model when using Spearman's rank correlation coefficients (purple) and based on the multiscale model (again using Spearman's rank correlation coefficients) if all changes in abundance were completely accounted for by changes in multiscale land cover (blue).
**Figure S12.** Map of Greater Vancouver, British Columbia, Canada showing 1995 land cover and locations of 1997 and 2020 bird surveys.

## Data Availability

Data and code are available from Open Science Foundation https://doi.org/10.17605/OSF.IO/KP2SR (Eyster, 2024).
