# Supplementary material for: Space‐for‐time substitutions exaggerate urban bird–habitat ecological relationships
Source: J Anim Ecol. 2024 Nov 6;93(12):1854–67. doi: 10.1111/1365-2656.14194 (PMC11615266; doi:10.1111/1365-2656.14194)

SUPPLEMENTARY INFORMATION FOR:

Space-for-time substitutions exaggerate urban bird–habitat ecological relationships

Harold N. Eyster[1,2,3,4,5*], Kai M. A. Chan[3,4], Morgan E. Fletcher[6], & Brian Beckage[1,2]
1. Gund Institute for Environment, University of Vermont, Burlington, VT, USA
2. Department of Plant Biology, University of Vermont, Burlington, VT, USA
3. Institute for Resources, Environment & Sustainability, University of British Columbia, Vancouver, BC, Canada
4. Biodiversity Research Centre, University of British Columbia, Vancouver, BC, Canada
5. Current affiliation: The Nature Conservancy in Colorado, Boulder, Colorado, USA
6. Rubenstein School of Environment and Natural Resources, University of Vermont, Burlington, VT, USA
* To whom correspondence should be directed; haroldeyster@gmail.com

Table S1: Optimal scales for each species and each land cover type found using boosted regression trees. Scales are radii measured in meters.

species	water	buildings	paved	bare	grass.herb	coniferous	Deciduous
amcr	250	100	850	1500	700	1500	350
amgo	350	1000	50	250	300	1200	200
amro	1500	1350	500	150	1500	400	100
anhu	1350	500	1500	1450	250	350	450
baea	1300	50	1100	950	50	650	1350
bars	300	50	50	1100	200	1450	50
bcch	1450	100	1500	1500	50	1300	200
bewr	250	350	450	1500	50	250	150
bhco	1300	150	900	1500	200	1450	100
bhgr	300	100	200	550	1050	50	50
blsw	1500	50	650	1000	1500	800	950
brcr	350	150	50	1500	1500	200	50
btyw	1400	100	450	1200	300	250	100
bush	1000	50	50	1100	50	450	200
cang	900	150	100	1200	700	800	200
cbch	1400	200	50	850	300	250	50
cedw	1500	100	750	1400	1450	250	100
chsp	200	1300	100	250	1500	50	100
coha	150	1500	700	750	1500	1450	50
cora	1250	100	1500	100	250	150	1450
coye	1000	350	1200	900	600	50	100
dowo	1500	250	850	1500	500	900	100
eucd	1500	1500	50	1250	100	200	250
eust	750	50	50	750	1500	800	650
gcki	700	50	750	950	1500	50	50
gwgu	900	150	100	1200	700	800	200
hofi	750	600	150	750	200	350	1500
hosp	1000	1500	50	1350	600	450	1500
huvi	1450	450	750	500	150	200	550
merl	150	1500	700	750	1500	1450	50
nrws	700	450	100	50	1500	250	100
ocwa	1200	900	100	1500	50	50	250
orju	1250	150	50	1500	350	750	50
pawr	1450	150	50	850	300	300	100
pisi	1250	1050	1500	1200	500	200	1000
piwo	1300	150	50	50	1200	200	100
psfl	750	100	450	200	50	350	50
pufi	750	600	150	750	200	350	1500
puma	1400	400	50	1150	550	950	250
rbgu	300	1500	1500	100	950	50	50
rbnu	1500	250	50	1450	100	150	150
rbsa	1500	250	850	1500	500	900	100

recr	700	50	50	500	1350	150	700
revi	950	300	1050	1200	50	250	100
ropi	950	250	250	550	150	900	1050
rsfl	1050	50	1500	1250	700	350	1500
ruhu	150	250	100	100	50	1450	50
savs	200	1300	100	250	1500	50	100
sosp	200	1300	100	250	1500	50	100
spto	900	400	900	50	50	600	100
stja	150	650	1200	900	750	150	50
swth	50	150	150	1450	50	250	50
vasw	1250	600	1500	750	1500	1450	50
vgsw	250	1500	1500	100	200	350	150
wavi	950	300	1050	1200	50	250	100
wcsp	1500	50	100	150	50	1300	100
weta	1050	300	400	250	50	1150	50
wewp	250	1500	100	950	1400	200	750
wifl	200	250	650	300	400	50	50
wiwa	350	100	100	50	1400	350	250
yrwa	250	1400	150	100	800	150	50

Table S2: Optimal scales for each species and each land cover type found using out-of-sample prediction and Pearson correlations. Scales are radii measured in meters.

Species	water	buildings	paved	bare	grass.herb	coniferous	Deciduous
amcr	1500	150	850	650	150	500	1500
amgo	50	500	650	500	50	1500	700
amro	500	900	500	1350	1300	1500	200
anhu	50	1400	1300	1500	950	1000	100
baea	150	250	650	50	100	650	900
bars	250	50	1500	50	100	250	200
bcch	1500	1500	1500	1500	1500	1500	50
bewr	1500	50	150	1500	50	50	900
bhco	1200	350	900	800	50	1500	50
bhgr	400	100	200	1200	800	900	150
blsw	1050	50	800	1500	250	1500	750
brcr	1500	150	150	1500	1500	150	50
btyw	250	150	400	250	350	850	200
bush	350	500	50	1500	450	50	50
cang	500	50	350	1250	100	250	50
cbch	50	150	100	1500	850	250	1500
cedw	1500	150	650	1300	1450	1500	100
chsp	1500	450	50	1250	800	1450	300
coha	550	150	150	50	50	100	50
cora	650	100	50	1500	700	100	1500

coye	1200	1500	1500	550	150	1500	250
dowo	1500	650	650	1500	550	1500	100
eucd	200	100	50	50	100	100	100
eust	550	1500	1500	50	50	300	50
gcki	1500	1500	1500	50	1500	50	1350
gwgu	1500	150	250	700	1050	400	1500
hofi	1500	1500	200	850	1500	650	1500
hosp	550	1500	1500	1350	200	550	100
huvi	900	650	750	350	200	350	750
merl	50	50	50	750	50	50	1500
nrws	1000	100	1500	950	1500	250	100
ocwa	1250	950	300	1400	1150	1500	550
orju	1500	350	400	1350	700	700	100
pawr	250	150	150	50	700	1000	50
pisi	1100	1500	1500	1500	50	1450	1350
piwo	900	1500	100	100	1500	300	100
psfl	650	150	500	1100	100	900	100
pufi	1200	700	950	1500	1400	1150	350
puma	600	500	50	50	650	650	950
rbgu	850	1500	1500	200	1100	50	1450
rbnu	1500	450	100	1500	850	200	400
rbsa	1250	1500	1500	1500	100	150	1500
recr	150	1050	1500	100	1050	1500	750
revi	1050	900	350	1050	450	50	500
ropi	200	150	300	150	150	400	1500
rsfl	50	100	1450	50	1500	700	250
ruhu	550	250	1500	950	50	1500	1500
savs	1450	50	100	1200	100	200	400
sosp	350	1400	100	1500	1500	1500	100
spto	750	450	650	700	50	1250	100
stja	500	700	750	200	300	100	50
swth	750	150	250	1150	100	1500	250
vasw	1500	600	150	100	1500	950	1500
vgsw	400	1500	1500	50	50	1500	250
wavi	900	250	450	1000	50	400	100
wcsp	1500	550	50	100	950	100	50
weta	1150	550	400	1300	450	1500	500
wewp	100	1400	250	1500	1200	250	1050
wifl	100	250	600	1200	350	1400	600
wiwa	50	800	300	1400	1050	1250	300
yrwa	750	50	150	150	50	100	1500

Table S3: Optimal scales for each species and each land cover type found using out-of-sample prediction and Spearman's correlations. Scales are radii measured in meters.

species	water	buildings	paved	bare	grass.herb	coniferous	Deciduous
amcr	1500	650	800	650	150	1500	800
amgo	1500	700	150	1300	250	1500	200
amro	1350	1050	1450	900	200	1500	500
anhu	1500	1350	1050	1500	550	1250	100
baea	1450	450	650	1450	100	650	150
bars	1500	50	1000	1500	250	1000	200
bcch	1500	1450	1450	900	1500	1500	250
bewr	1500	150	150	1500	50	300	150
bhco	1500	550	1450	700	150	1500	200
bhgr	550	150	50	550	800	850	100
blsw	1150	50	450	1050	300	1050	600
brcr	1500	150	150	1500	1500	350	150
btyw	1500	200	200	250	350	200	50
bush	1500	1450	50	1500	450	1450	150
cang	500	500	200	50	350	50	200
cbch	850	600	200	1500	850	200	1500
cedw	1300	700	650	900	1350	1500	300
chsp	1500	450	50	1250	750	650	1500
coha	550	450	50	350	200	50	50
cora	650	100	50	1500	450	50	1500
coye	1200	1500	1500	150	150	1400	50
dowo	1500	700	650	1500	500	1450	100
eucd	1500	100	50	1450	150	100	100
eust	1500	50	1200	1500	800	650	1500
gcki	1500	1500	1500	1500	1500	50	1350
gwgu	1300	150	1100	900	1050	400	150
hofi	1500	800	200	1500	1500	700	1500
hosp	1500	150	1150	700	150	500	1500
huvi	900	650	750	350	450	300	650
merl	1500	50	50	750	50	50	1500
nrws	750	100	1500	950	1500	150	100
ocwa	1200	1150	300	1500	1150	1400	1300
orju	1500	450	400	1400	1000	1500	150
pawr	250	100	100	1500	800	250	50
pisi	1400	1200	700	1500	850	1500	600
piwo	900	1500	100	150	1500	100	100
psfl	1200	600	500	650	250	400	50
pufi	1200	750	950	1500	1450	1050	350
puma	1450	650	50	1500	500	1000	950
rbgu	650	1500	1500	50	1100	50	950
rbnu	1500	450	150	1500	50	150	300
rbsa	750	50	100	50	200	150	150

recr	1500	1050	1500	1500	1050	200	800
revi	1050	100	400	1050	450	50	400
ropi	1200	250	750	1500	250	400	1250
rsfl	1500	100	1000	1300	1500	1500	300
ruhu	1500	300	1500	100	50	1350	1500
savs	1450	50	150	1200	500	200	150
sosp	450	1000	200	1350	1500	100	100
spto	800	500	650	650	250	600	100
stja	700	750	750	200	1500	100	50
swth	1300	150	200	1000	750	1450	100
vasw	1500	600	150	1250	900	950	1500
vgsw	1500	1400	1500	350	250	1300	500
wavi	1200	200	350	1500	50	250	100
wcsp	1500	1250	50	400	100	1500	50
weta	1150	1350	250	800	450	1500	1500
wewp	1450	250	250	1500	1200	200	100
wifl	50	250	600	700	400	1150	600
wiwa	350	450	150	700	850	900	300
yrwa	550	50	100	1450	50	150	1500

Table S4: Estimates of true abundance (medians and 89% CrI)

species	1997 med.	2020 med.	1997 lower	1997 upper	2020 lower	2020 upper
amcr	605	551	518	710	475	634
amgo	4	73	0	10	50	101
amro	331	168	239	424	127	218
anhu	1	128	0	5	94	167
baea	0	2	0	2	0	6
bars	123	24	69	201	10	41
bcch	347	586	291	407	512	657
bewr	1	23	0	5	10	37
bhco	81	12	39	135	4	24
bhgr	2	8	0	7	1	16
blsw	2	50	0	7	23	84
brcr	1	14	0	3	6	25
btyw	1	13	0	3	5	23
bush	150	198	119	188	162	231
cang	6	0	1	15	0	3
cbch	1	54	0	3	38	73
cedw	50	126	20	92	66	199
chsp	0	4	0	3	0	9
coha	0	1	0	2	0	4
cora	1	7	0	3	1	14
coye	2	0	0	7	0	3

dowo	1	6	0	3	0	13
eucd	1	6	0	3	1	14
eust	1000	205	835	1181	164	250
gcki	1	7	0	3	2	16
gwgu	193	60	146	255	40	81
hofi	445	298	358	537	240	355
hosp	954	88	829	1108	67	113
huvi	0	2	0	2	0	6
merl	0	3	0	3	0	8
nrws	0	4	0	3	0	10
ocwa	2	8	0	6	2	16
orju	25	32	11	44	16	50
pawr	3	10	0	8	3	18
pisi	10	138	3	19	108	172
piwo	0	2	0	2	0	7
psfl	1	67	0	3	47	88
pufi	0	2	0	2	0	6
puma	2	34	0	7	16	66
rbgu	1	12	0	5	4	22
rbnu	2	44	0	7	27	63
rbsa	0	1	0	2	0	3
recr	2	40	0	7	16	74
revi	2	1	0	7	0	4
ropi	269	117	215	333	89	146
rsfl	1	50	0	4	32	70
ruhu	12	1	2	24	0	3
savs	0	1	0	2	0	3
sosp	46	96	28	67	73	122
spto	128	81	90	168	60	108
stja	6	9	0	13	2	18
swth	49	60	31	75	41	86
vasw	2	48	0	8	21	85
vgsw	358	155	256	456	115	205
wavi	1	26	0	4	13	42
wcsp	49	109	30	72	81	140
weta	1	9	0	3	2	19
wewp	0	5	0	3	1	11
wifl	11	4	3	23	1	11
wiwa	1	15	0	4	6	28
yrwa	0	4	0	3	1	10

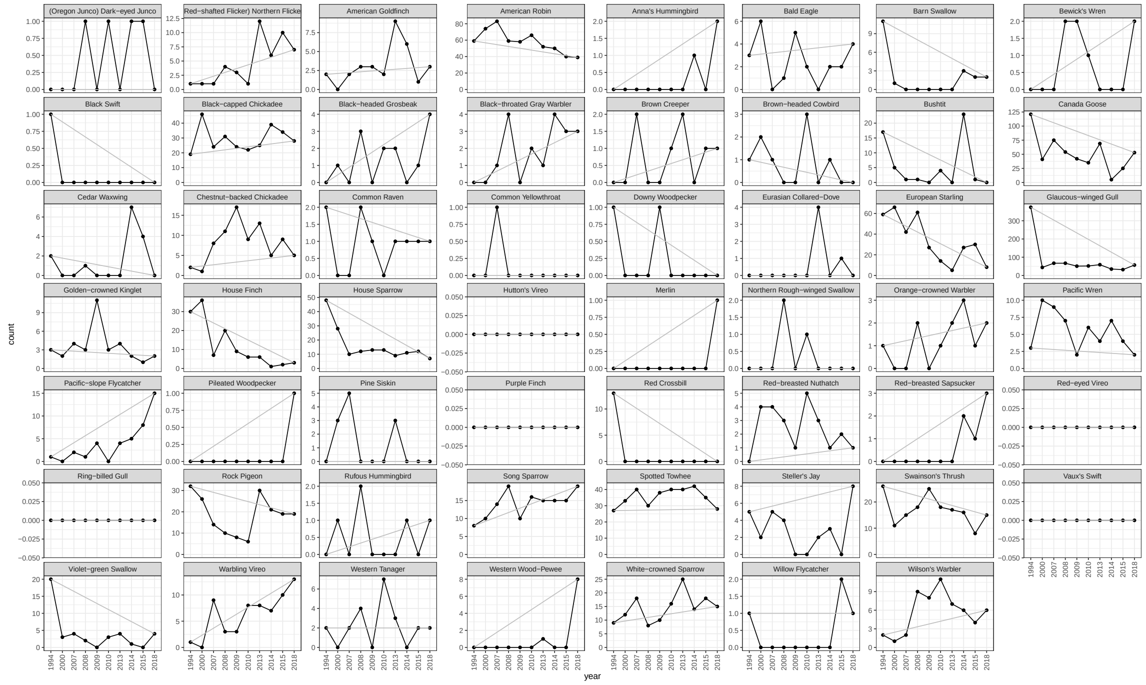

Figure S1: Plots of birds contained in our datasets that were also detected by the North American Breeding Bird Survey route 11-212. Gray lines show trends estimated just using the 1994 and 2018 endpoints. Black lines and points show detections for all ten years of observations. Data from Ziolkowski et al. (2023).

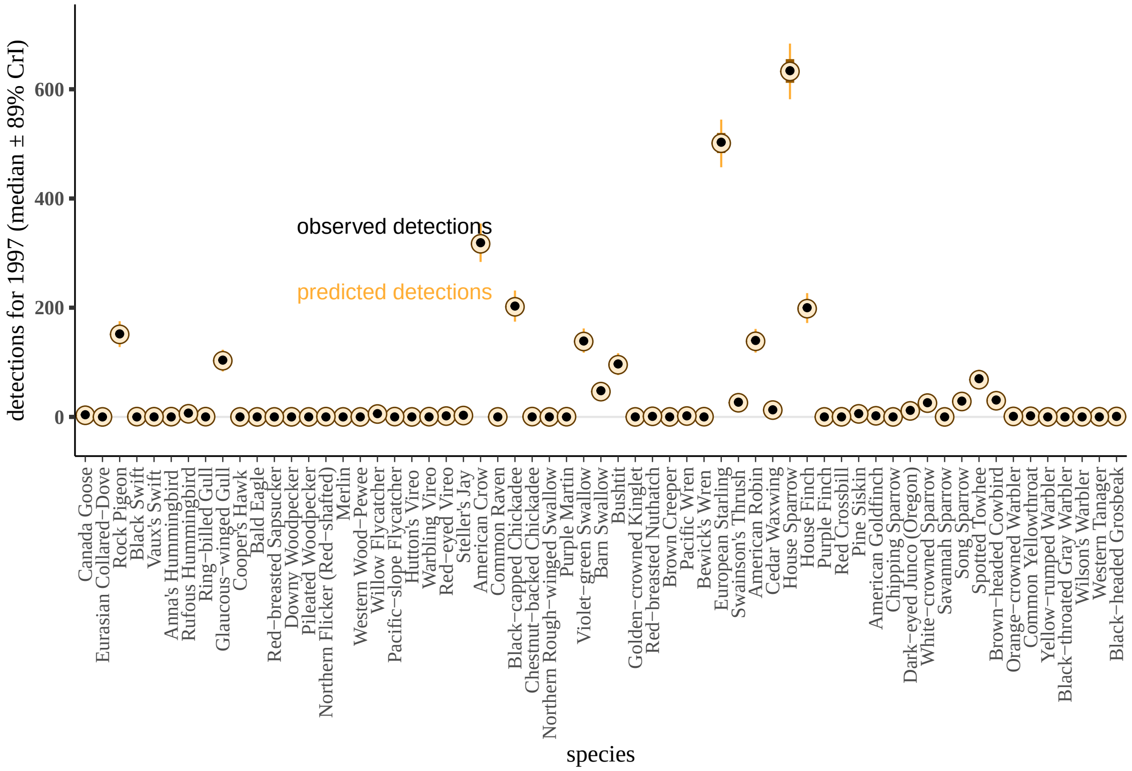

Figure S2: Total observed (black dots) and modeled (orange circles and 89% credible intervals) detections of each species in 1997.

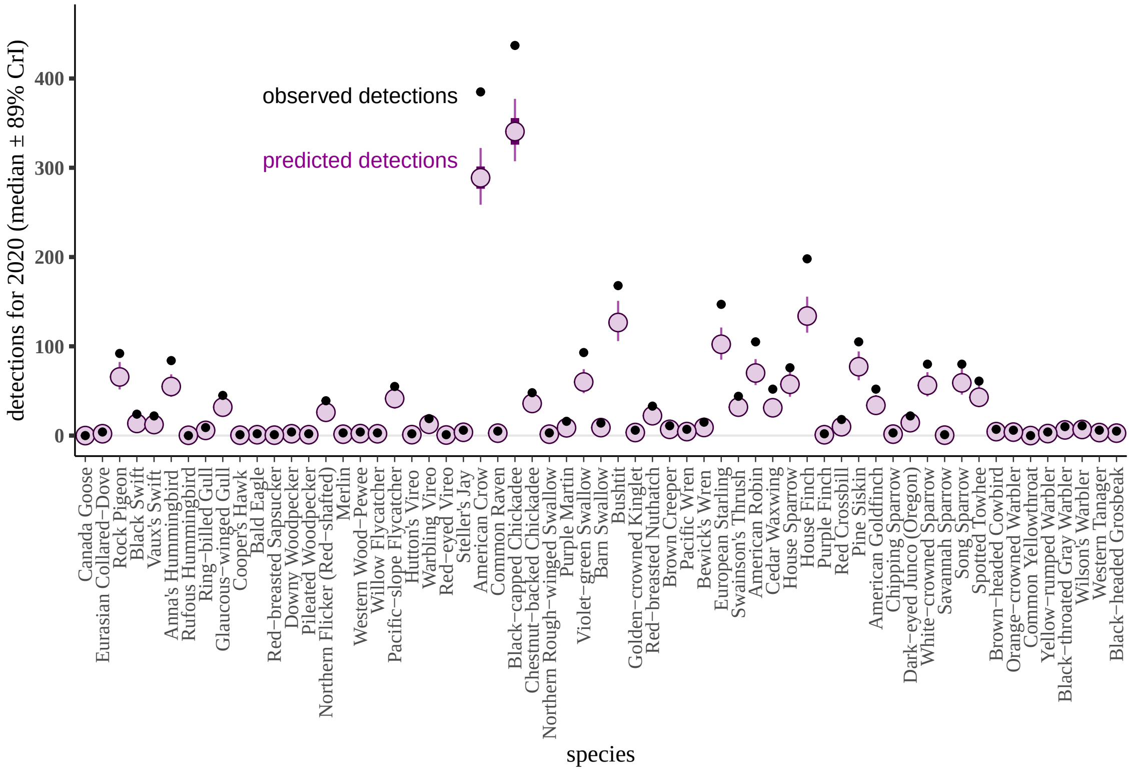

Figure S3: Total observed (black dots) and modeled (purple circles and 89% credible intervals) detections of each species in 2020.

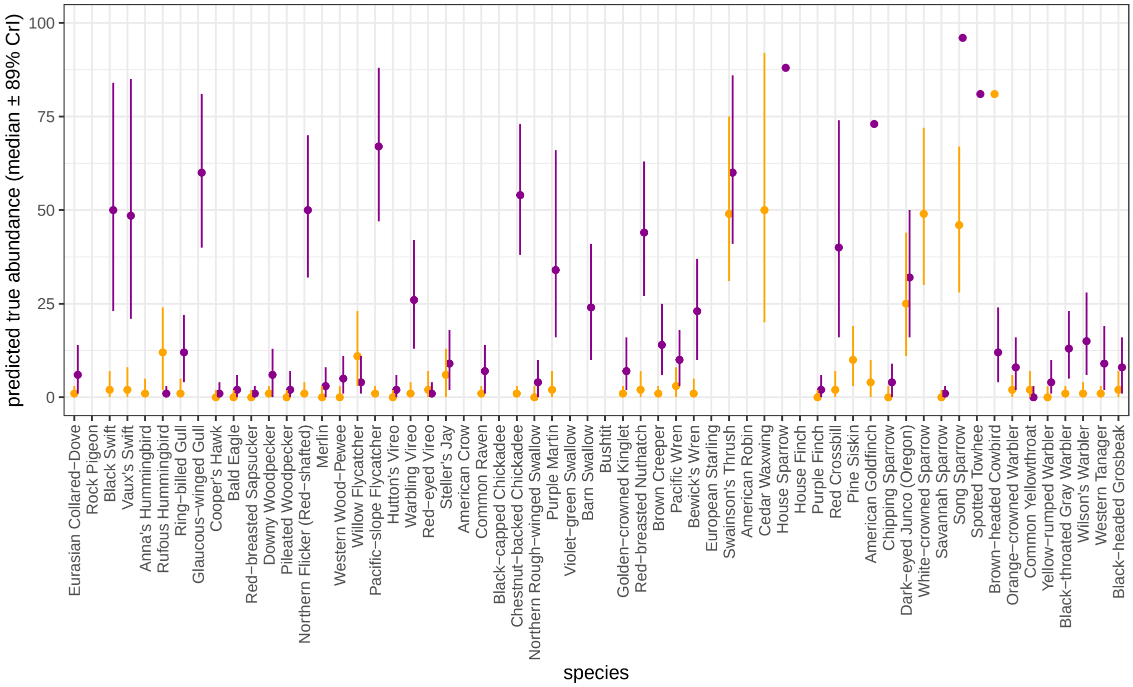

Figure S4: Estimated true abundance of less-abundant species across all sampling sites in 1997 (orange) and 2020 (purple) based on the multiscale model using boosted regression trees to estimate optimal spatial scales. 89% credible intervals are shown. To see abundance trends of all species, see Fig. 2.

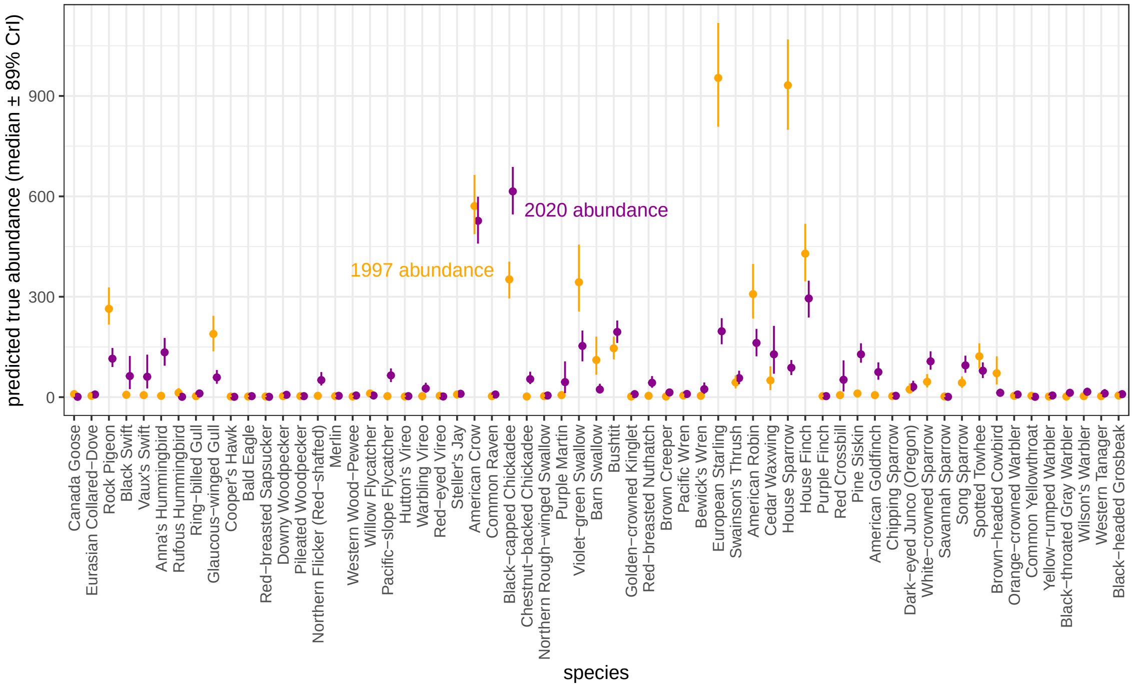

Figure S5: Estimated true abundance of each species across all sampling sites in 1997 (orange) and 2020 (purple) based on the 50-m model. 89% credible intervals are shown.

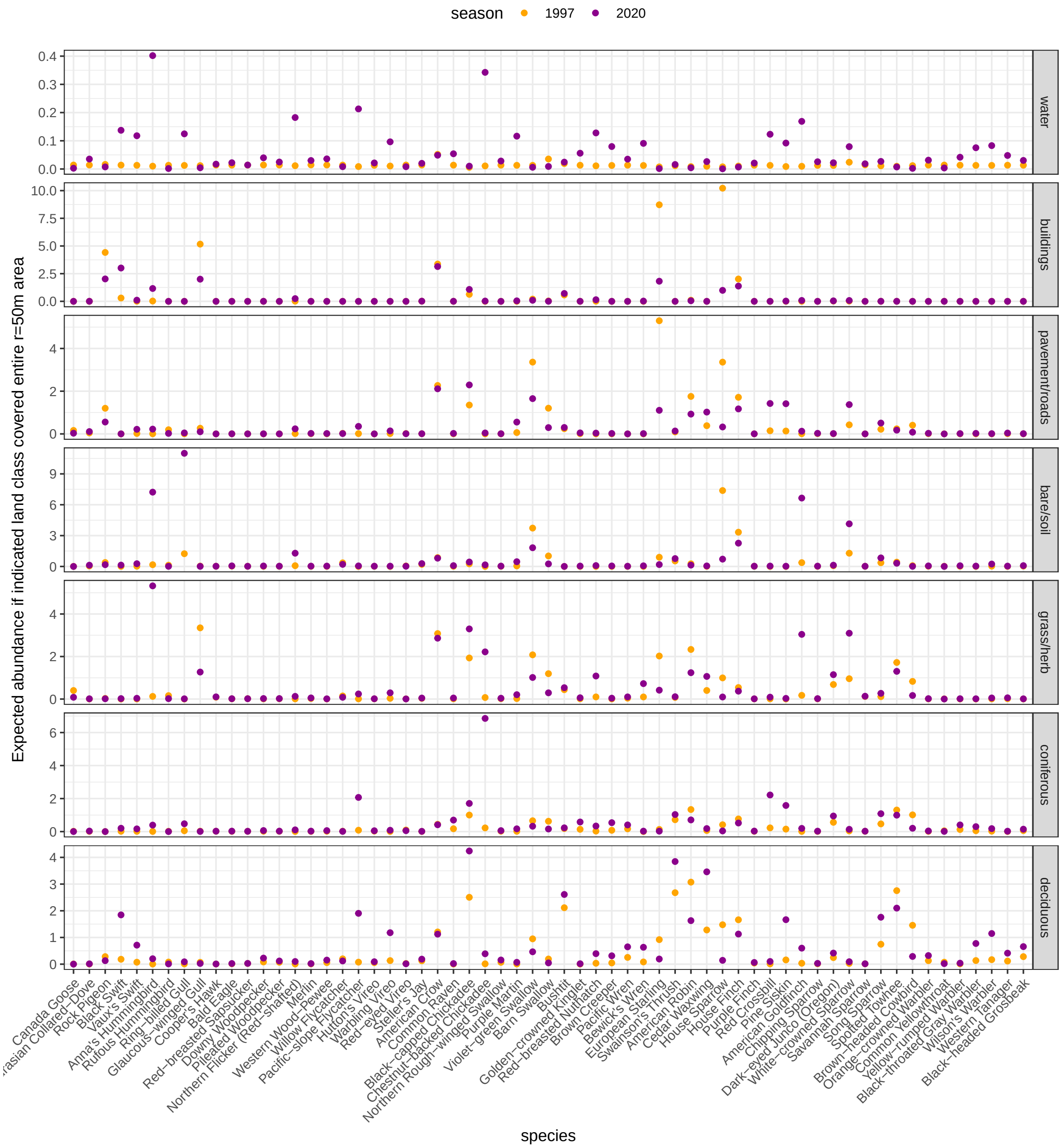

Figure S6: Estimated true abundance of each species if the entire 50-m area surrounding each sampling point consisted of the indicated habitat type in 1997 (orange) and 2020 (purple). 89% credible intervals are shown.

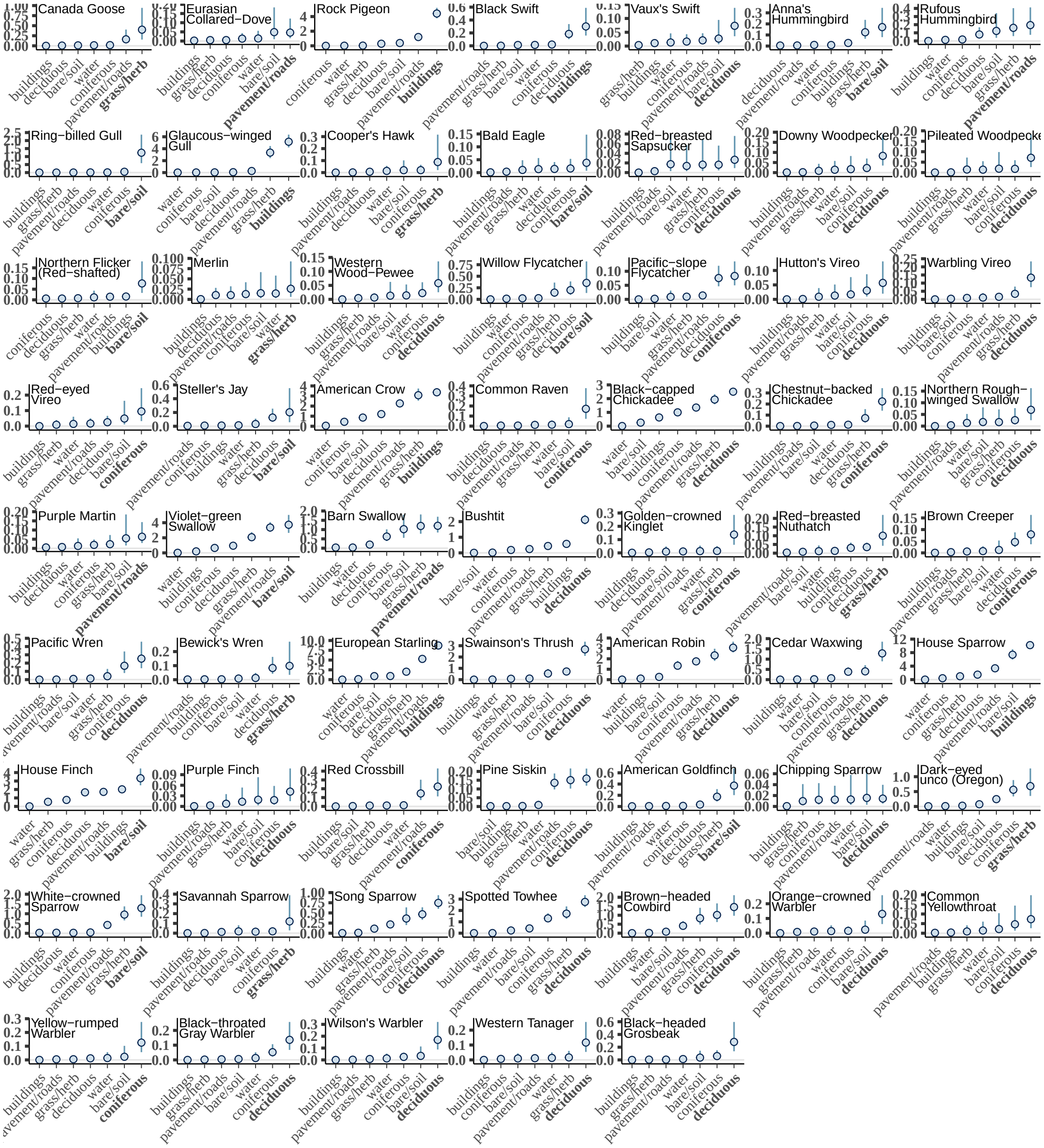

Figure S7: Modeled abundance of each species in 1997 if the entire 50-m site was occupied by the indicated land cover type. For each species, land cover types are ordered by associated abundance (the type associated with the greatest abundance is bolded). 89% credible intervals are shown.

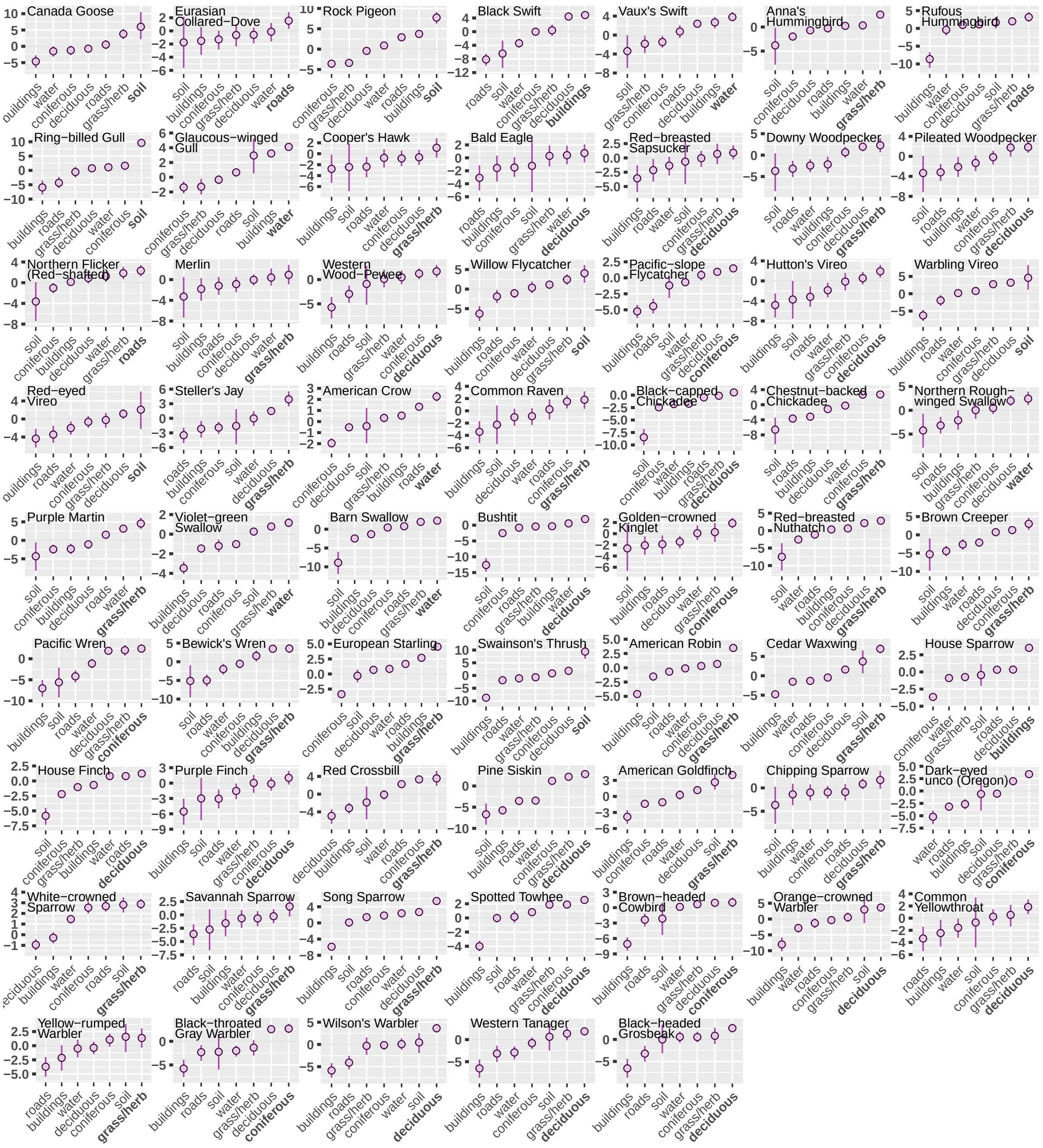

Figure S8: Estimated effects of each habitat type when measured at the optimum scale (identified using boosted regression trees) for each species in 1997 (orange) and 2020 (purple). 89% credible intervals are shown.

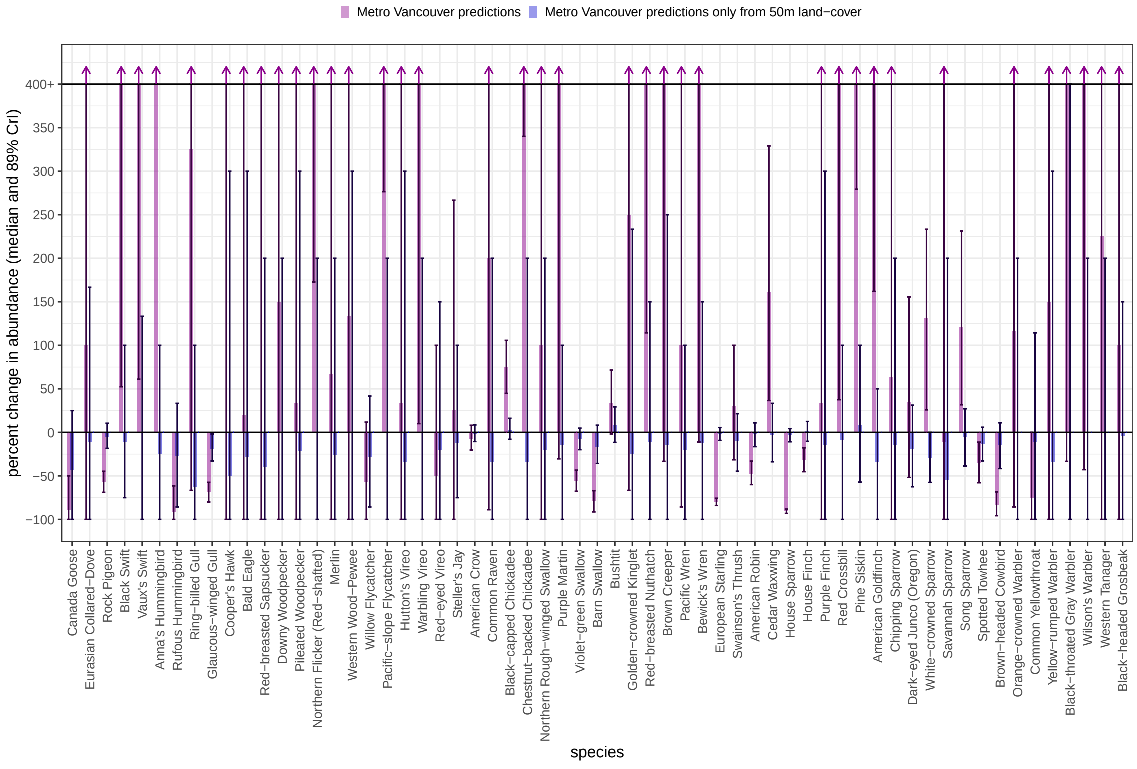

Figure S9: Percent change in abundance of each bird species across greater Vancouver 1997–2020 based on the 50 m landcover model (purple) and based on the 50 m landcover model if all changes in abundance were completely accounted for by changes in landcover within the 50-m radius point count (blue). Note that medians and error bars extending beyond 400% are indicated with an arrow. Note that data for Eurasian Collared-Dove was not available across Canada. National trends for American Crow are given by national trends for Northwestern Crow. Medians and 89% credible intervals are shown.

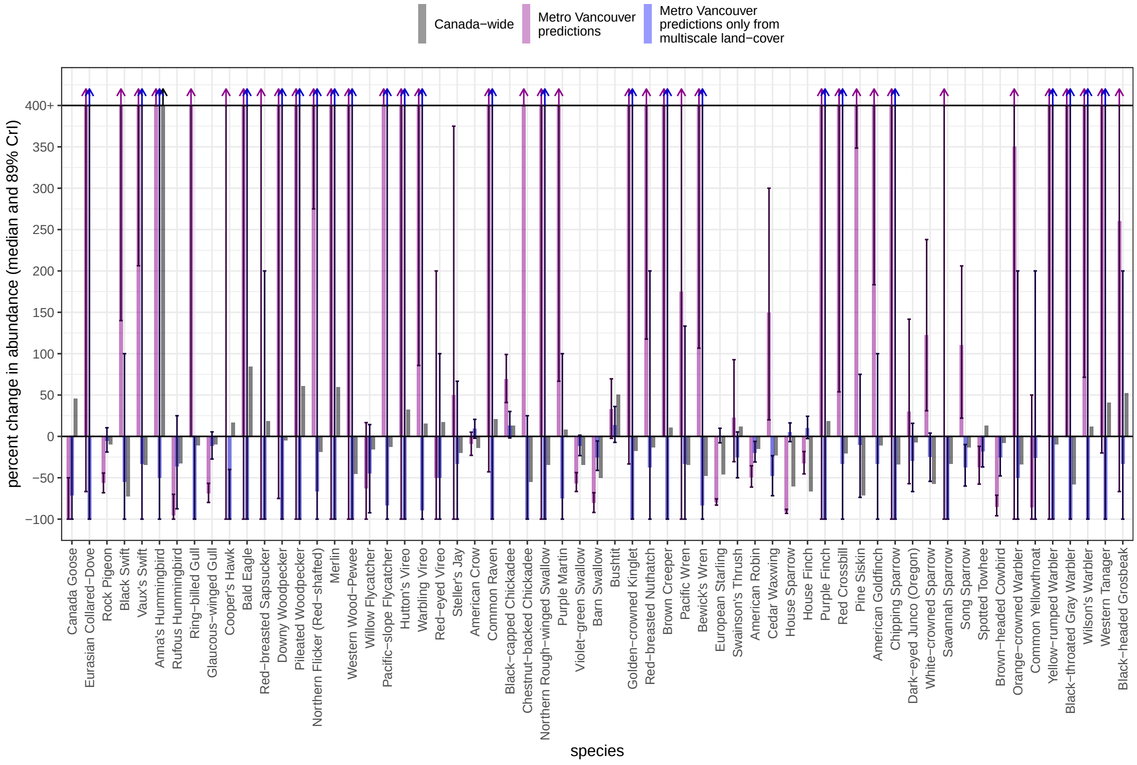

Figure S10: Percent change in abundance of each bird species across Canada 1993–2016 (gray; data from North American Bird Conservation Initiative Canada (2019)), across greater Vancouver 1997–2020 based on the multiscale model using Pearson correlations (purple) and based on the multiscale model (again using Pearson correlations) if all changes in abundance were completely accounted for by changes in multiscale land cover (blue). Note that data for Eurasian Collared-Dove was not available across Canada. National trends for American Crow are given by national trends for Northwestern Crow. Note that medians and error bars extending beyond 400% are indicated with an arrow. Medians and 89% credible intervals are shown.

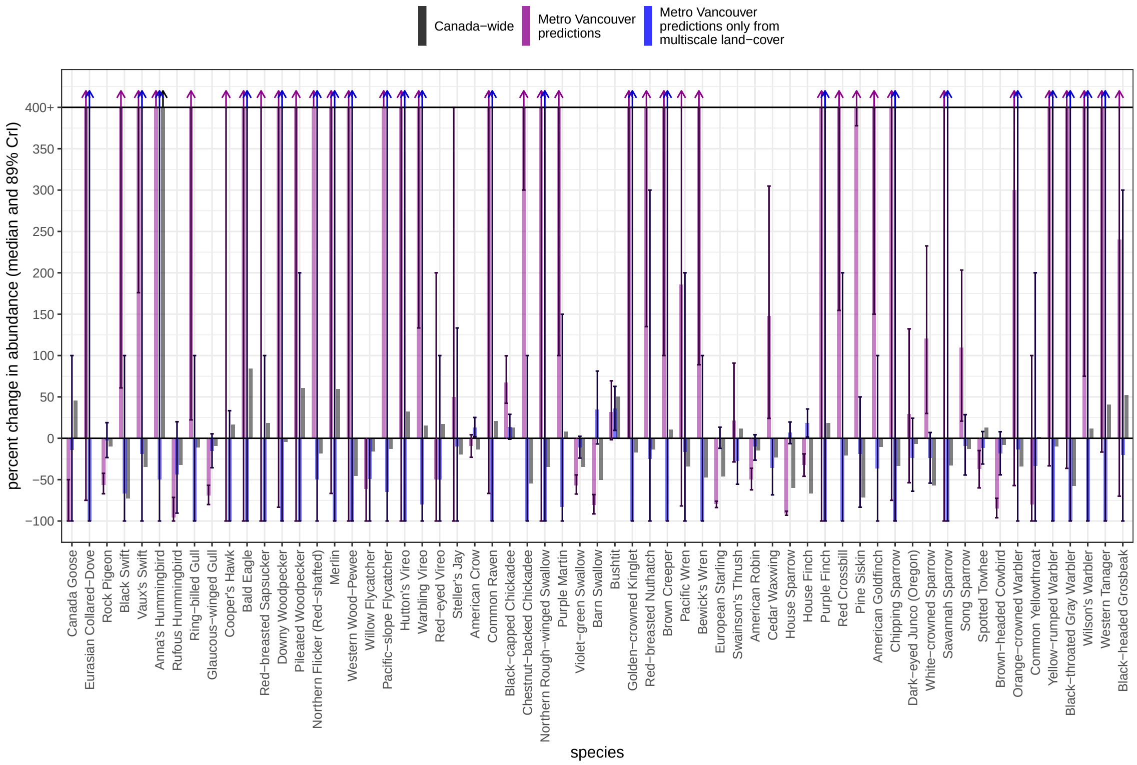

Figure S11: Percent change in abundance of each bird species across Canada 1993–2016 (gray; data from North American Bird Conservation Initiative Canada (2019)), across greater Vancouver 1997–2020 based on the multiscale model when using Spearman's rank correlation coefficients (purple) and based on the multiscale model (again using Spearman's rank correlation coefficients) if all changes in abundance were completely accounted for by changes in multiscale land cover (blue). This model mostly performs worse than the one that selected scales using Pearson correlations (see e.g., Barn Swallow; cf. Bushtit). Note that data for Eurasian Collared-Dove was not available across Canada. National trends for American Crow are given by national trends for Northwestern Crow. Note that medians and error bars extending beyond 400% are indicated with an arrow. Medians and 89% credible intervals are shown.

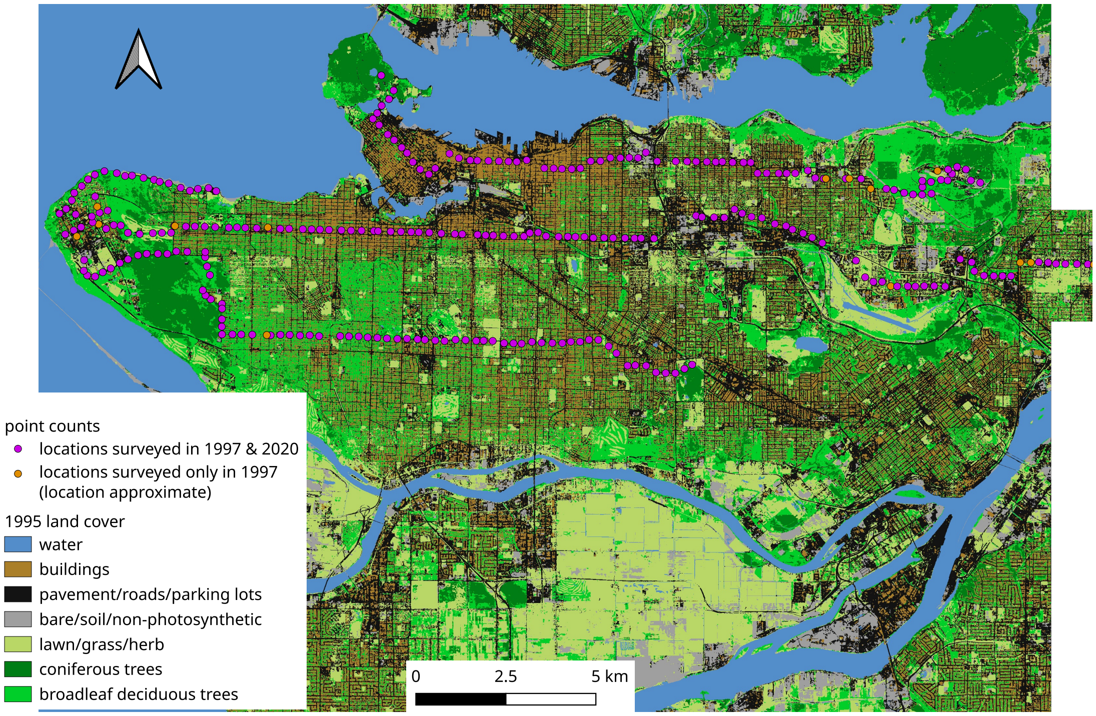

Figure S12: Map of Greater Vancouver, British Columbia, Canada showing 1995 land cover and locations of 1997 and 2020 bird surveys. For 2021 landcover map, see Fig. 1.

Species-specific results and management implications

Some species exhibited lower abundance in 2020 than 1997, including Canada Goose, Rock Pigeon, Rufous Hummingbird, Glaucous-winged Gull, Violet-green Swallow, Barn Swallow, European Starling, American Robin, House Sparrow, House Finch, Spotted Towhee, Brown-headed Cowbird (Fig. 3).
Many other species exhibited higher abundance in 2020 than 1997, including Black Swift, Vaux's Swift, Anna's Hummingbird, Ring-billed Gull, Northern Flicker, Pacific-slope Flycatcher, Warbling Vireo, Black-capped Chickadee, Chestnut-backed Chickadee, Purple Martin, Red-breasted Nuthatch, Brown Creeper, Bewick's Wren, Cedar Waxwing, Red Crossbill, Pine Siskin, American Goldfinch, White-crowned Sparrow, Song Sparrow, Black-throated Gray Warbler, Wilson's Warbler (Fig. 3). Although their 89% CrI crossed zero, American Crow may have declined, while Bushtit and Western Tanager may have increased (Fig. 3).
Some of these changes in abundance were consistent with national changes in bird abundance, such as Anna's Hummingbird, American Crow, Bushtit, and Western Tanager. Other local trends were opposite to national trends. For example, Cedar Waxwing increased in Metro Vancouver but decreased nationally; Spotted Towhee decreased in Metro Vancouver but increased nationally. Other trends were more pronounced in Metro Vancouver than nationally. For example, House Sparrow, European Starling, and Rock Pigeon declined locally more than nationally. Similarly, Purple Martin and Black-capped Chickadees increased locally more than nationally (Fig. 3).
Our results have important implications for urban forest management. Conifers and broadleaf trees supported different bird communities. Some species were much more abundant in conifers, such as Golden-crowned Kinglet, Brown Creeper, and Chestnut-backed Chickadee, while others were much abundant near broadleaf trees, such as Downy Woodpecker, Orange-crowned Warbler and Wilson's Warbler (Fig. S7). These results show the importance of differentiating between different types of tree cover in analyses, and the large avian impacts of the City of Vancouver's decision to primarily plant broadleaf trees along its streets (City of Vancouve Open Data Portal, 2022; Ley, 1995). Still other species preferred areas with fewer trees, for example Northern (Red-shafted) Flickers were more abundant near bare soil, Savannah Sparrows were most abundant near grass/herb, Eurasian Collared Doves were most abundant near pavement/roads, and European Starlings were most abundant near buildings (Fig. S7). Urban forest management decisions will require careful considerations about which bird communities are most important to support.

Point count datasheet template

Point Information

Point ID: _______
Observer:___________________
Year: _________
month: _________
day: _________
Start time: _________
Colors used for each period:
0-5 min: __________
5-10 min: ________

Weather

Cloud cover: _______ %
Precip: ________
Temp: _________°C
Wind: _____
0: Calm. Smoke rises vertically (<1 km/hr)
1: Smoke drifts. Leaves stationary(1-5 km/hr)
2: Wind felt on skin, leaves rustle (6-11 km/hr)
3: Leaves and twigs cosntantly move (12-19km/hr)
4: Small branches begin to move (20-28 km/hr

Instructions

- Count should be done in fair weather within four hours after sunrise.
- All birds seen or heard within the 50 meter readius count cirle should be recorded. See map to note landmarks that delimit the circle.
- BC Breeding code should also be included (see attached).
- Record bird behavior and height above ground (when initially observed)
- Record substrate type (e.g., white ash, power line), and substrate height.
- Alpha codes or full species name may be used.
- Record all birds detected during first 5 minutes using one color pen, and all birds during the second 5 minutes using a different color. If a bird is observed during both periods, record it in both colors.
- If a bird is flying above the circle (e.g., a gull), enter it in the 'flyover' box. However, if it is foraging/using the habitat (E.g., swifts, swallows, rapters), then record it within the circle .
-Record cars/peds/bikes within circle. If there are many cars, extropolate.

Traffic & Noise

Pedestrians #:
Cars (estimate)#:
Bikers #:
Dogs #:
Other noise present:
How much did noise obscure your hearing?
(circle one): **1 2 3 4 5 6 7 8 9 10**

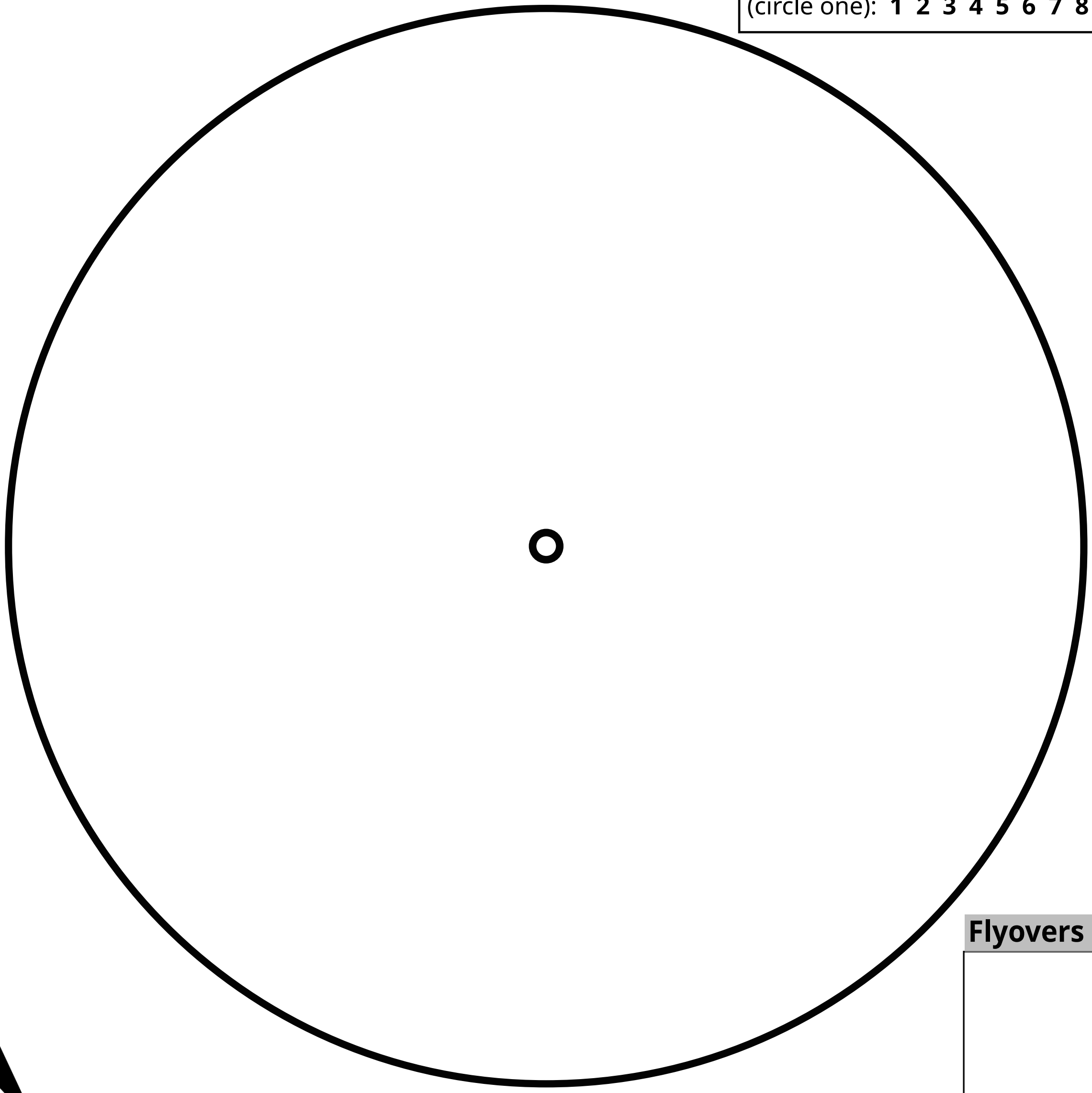

50 meter radius

Flyovers

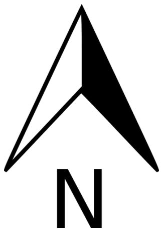

Supplement: Supplementary file 1 — Table S1. Optimal scales for each species and each land cover type found using boosted regression trees. Table S2. Optimal scales for each species and each land cover type found using out‐of sample prediction and Pearson correlations. Table S3. Optimal scales for each species and each land cover type found using out‐of sample prediction and Spearman's correlations. Table S4. Estimates of true abundance (medians and 89% CrI). Figure S1. Plots of birds contained in our datasets that were also detected by the North American Breeding Bird Survey route 11‐212. Figure S2. Total observed (black dots) and modeled (orange circles and 89% credible intervals) detections of each species in 1997. Figure S3. Total observed (black dots) and modeled (purple circles and 89% credible intervals) detections of each species in 2020. Figure S4. Estimated true abundance of less‐abundant species across all sampling sites in 1997 (orange) and 2020 (purple) based on the multiscale model using boosted regression trees to estimate optimal spatial scales. Figure S5. Estimated true abundance of each species across all sampling sites in 1997 (orange) and 2020 (purple) based on the 50‐m model. Figure S6. Estimated true abundance of each species if the entire 50‐m area surrounding each sampling point consisted of the indicated habitat type in 1997 (orange) and 2020 (purple). Figure S7. Modeled abundance of each species in 1997 if the entire 50‐m site was occupied by the indicated land cover type. Figure S8. Estimated effects of each habitat type when measured at the optimum scale (identified using boosted regression trees) for each species in 1997 (orange) and 2020 (purple). Figure S9. Percent change in abundance of each bird species across greater Vancouver 1997–2020 based on the 50 m landcover model (purple) and based on the 50 m landcover model if all changes in abundance were completely accounted for by changes in landcover within the 50‐m radius point count (blue). Figure S10. Percent change i [file JANE-93-1854-s001.pdf]
